# Red Blood Cell‐Induced Bacterial Margination Improves Microbial Hemoadsorption on Engineered Cell‐Depleted Thrombi, Restoring Severe Bacteremia in Rats

**DOI:** 10.1002/advs.202417498

**Published:** 2025-04-26

**Authors:** Bong Hwan Jang, Su Hyun Jung, Seyong Kwon, Sung Jin Park, Joo H. Kang

**Affiliations:** ^1^ Department of Biomedical Engineering College of Information and Biotechnology Ulsan National Institute of Science and Technology (UNIST) UNIST gil 50 Ulsan 44919 Republic of Korea

**Keywords:** bacteremia, bacterial adhesin receptors, bacterial margination, blood cell‐depleted thrombus, extracorporeal blood cleansing, sepsis treatment

## Abstract

Extracorporeal hemoadsorption for treating bacteremia has exhibited limited success due to the lack of a clear strategy for effectively bringing bacterial cells into contact with the surface and universal bacteria‐capturing substances. Here, a novel extracorporeal device is reported that can eliminate various intact bacteria from whole blood, employing microfluidic bacterial margination and engineered cell‐depleted thrombus (CDT) presenting bacterial adhesin receptors. The critical strain rate of red blood cells (RBCs) (0.83 × 10^−2^) and the flow path height within about 300 µm required for RBC axial migration in the flows are found. The subsequent RBC‐bacteria collisions induced bacterial margination, facilitating their effective capture on the CDT surface on the channel wall. Fibrinogen and fibronectin in CDT are found to primarily contribute to capturing various bacteria. The extracorporeal CDT filters (eCDTF), which integrate all these principles, demonstrate significant depletion of major antibiotic‐resistant and human fecal bacteria from the whole blood in vitro. Remarkable reductions in bacterial load and inflammatory markers in the rats lethally infected with methicillin‐resistant *Staphylococcus aureus* are further confirmed, resulting in the restoration from bacteremia following extracorporeal treatment. The demonstration may propose a new design principle for hemoadsorption devices and elucidate the limited success of conventional treatments.

## Graphical abstract

A highly efficient extracorporeal device for treating bacteremia is developed by integrating microfluidic bacterial margination and engineered cell‐depleted thrombi strategically constructed in the device. The rodent models, severely infected with antibiotic‐resistant bacteria, recover from bacteremia after two subsequent extracorporeal blood treatments. This approach offers new insight into the design of hemoadsorption devices, overcoming the limitations of conventional extracorporeal therapies.

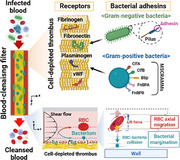

## 1 Introduction

Bacteria from localized infections, such as urinary tract infections, pneumonia, or infected wounds, can enter the bloodstream, potentially leading to bacteremia. If left untreated or under certain conditions, bacteremia could progress to bloodstream infections (BSI), infective endocarditis, and immunological complications, particularly in immunocompromised patients, such as those undergoing chemotherapy, organ transplantation, or premature infants.^[^ ^1^, ^2^, ^3^, ^4^ ^]^ When a BSI triggers an excessive inflammatory response, it can damage blood vessels and impair tissue and organ function, a hallmark of sepsis. Furthermore, BSI is a life‐threatening condition with high mortality rates (14–37%), in part because of time‐consuming and often low‐sensitivity nature of bacteria detection in the bloodstream.^[^ ^5^, ^6^ ^]^ In 2019, 7.7 million deaths worldwide were attributed to 33 bacterial pathogens, including both antimicrobial‐resistant and ‐susceptible strains.^[^ ^7^ ^]^ Moreover, the widespread empirical use of broad‐spectrum antibiotics can contribute to the emergence of antibiotic‐resistant bacteria, such as carbapenem‐resistant *Enterobacteriaceae* (CRE) and multidrug‐resistant *Pseudomonas aeruginosa* (MRPA).^[^ ^8^, ^9^ ^]^

To overcome the drawbacks associated with previous sepsis guidelines, researchers have emphasized the need to eliminate a source of microbial infection from the entire bloodstream. These interests recently have merged into extracorporeal blood‐cleansing approaches, which can remove a broad range of pathogens without requiring time‐consuming prior diagnosis. Several previous endeavors have demonstrated that the application of extracorporeal devices, reducing pathogens or endotoxin in the blood, can enhance the clinical outcomes of patients afflicted with bacteremia or endotoxemia.^[^ ^10^ ^]^ The interior surface of those fluidic channels was functionalized by integrating synthetic proteins or antibiotics. This advanced hemofilter demonstrated its efficacy by successfully eliminating free lipopolysaccharides (LPS) and pathogen‐associated molecular patterns (PAMPs) from the blood through hemoadsorption.^[^ ^11^ ^]^ However, the current design is insufficient for targeting intact bacteria due to the absence of an effective strategy to increase bacterial contact with the functionalized surface within the fluidic channel. Moreover, a single set of functional materials, such as polymyxin B or polyethyleneimine (PEI), covers a limited range of target bacterial ligands.^[^ ^12^, ^13^ ^]^ Due to these limitations and inconclusive evidence, the recent guideline suggests against the use of hemoperfusion for treating sepsis.^[^ ^14^ ^]^ Despite the technical limitation, there were sparse research outcomes that support the conventional hemoadsorption device's ability to remove intact bacteria in the whole blood.^[^ ^15^, ^16^ ^]^ However, detailed mechanisms explaining the limited success of hemoadsorption treatment in certain clinical settings have not been elucidated.

Here, we biologically inspired a hemoadsorption principle that effectively eliminates a broad range of intact bacteria in whole blood by incorporating red blood cell (RBC) deformability‐driven bacterial margination and cell‐depleted thrombus strategically constructed in an extracorporeal device. Due to their inherent low diffusion coefficients of intact bacteria in blood, we leveraged the axial migration of RBCs that promotes outward repelling of bacteria due to collisions between RBCs and bacteria, which drives bacteria to move away from centered RBCs streamlines toward the thrombus surface where a broad range of bacteria can be captured by plasma proteins, including fibrinogen, fibronectin, plasminogen, and von Willebrand factor (vWF). This approach effectively removed a wide variety of bacteria, including Gram‐positive, Gram‐negative, and even antibiotic‐resistant strains, from the whole blood. The rodent animal models severely challenged with bacteria were also successfully recovered after the extracorporeal blood‐cleansing treatment.

## 2 Results

### 2.1 Universal Bacteria‐Capturing Thrombus Surfaces

**Figure 1:**
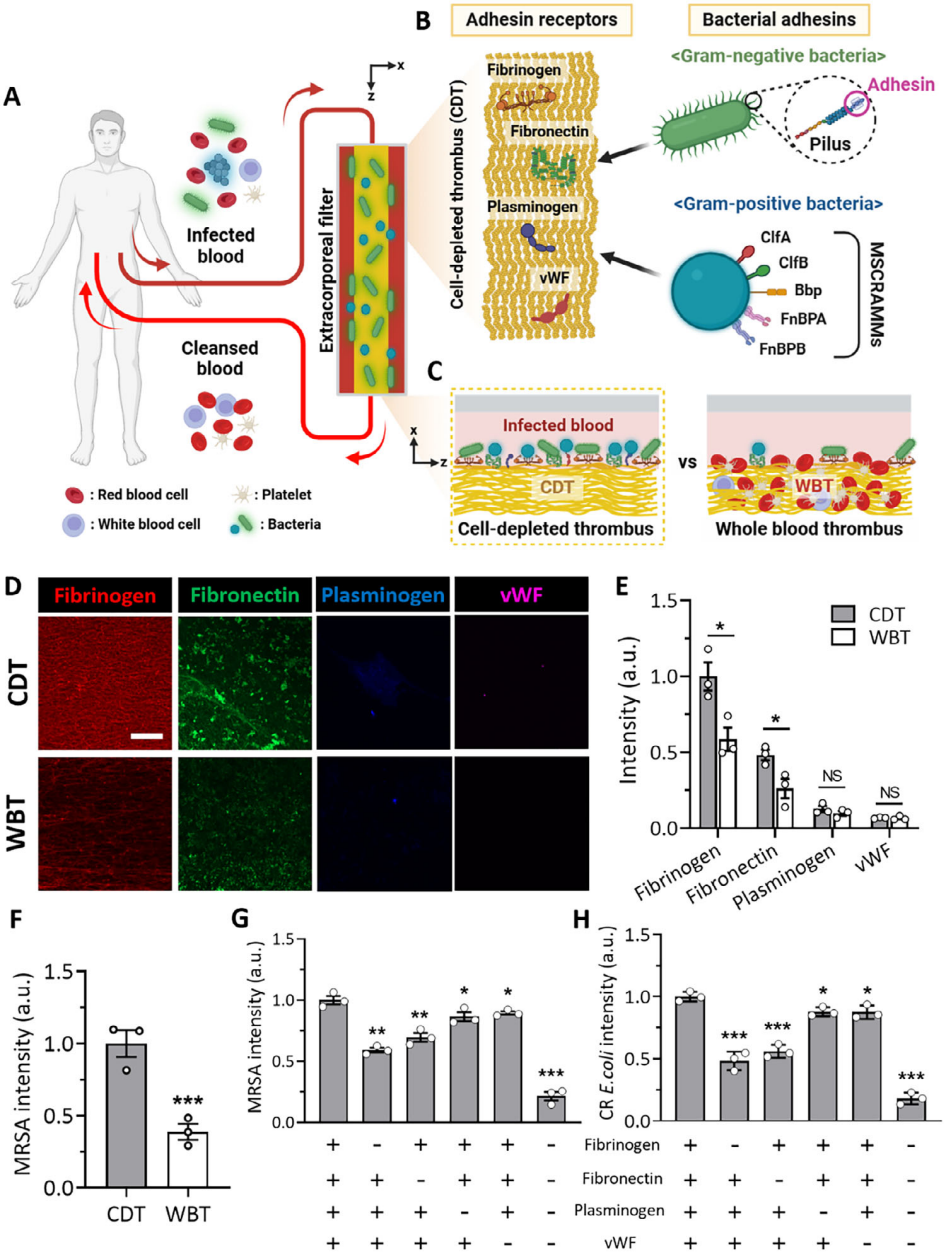
The universal bacterial‐capturing capability of adhesin receptor proteins on the cell‐depleted thrombus (CDT). A) The extracorporeal blood‐cleansing system through hemoadsorption onto the functional CDT surface. B) The CDT comprises the adhesin receptor proteins, including fibrinogen, fibronectin, plasminogen, and von Willebrand factor (vWF), which bind bacterial pili of Gram‐negative bacteria and microbial surface components recognizing adhesive matrix molecules (MSCRAMMs) of Gram‐positive bacteria. C) Thrombi from platelet‐poor plasma are expected to capture bacteria more effectively, whereas blood cells (mostly RBCs and platelets) occupy most of the surface of whole blood thrombus (WBT), thereby inhibiting interactions between adhesin receptor proteins and various bacteria. D) Immunofluorescence staining of adhesin receptor proteins on the surface of CDT and WBT, highlighting the presence of fibrinogen (red), fibronectin (green), plasminogen (blue), and vWF (violet). Scale bar: 50 µm. E) Quantitative analysis reveals a higher abundance of adhesin receptor proteins on the CDT surface relative to that of WBT, with fibrinogen and fibronectin being notably prevalent in both thrombus types (*n* = 3). F) The abundant presence of adhesin receptor proteins on the CDT surface correlates with an enhanced bacterial (methicillin‐resistant *Staphylococcus aureus*, MRSA) binding efficiency in comparison to the WBT surface (*n* = 3). G,H) Bacteria depletion efficiencies of the CDT surfaces when adhesin receptor proteins (fibrinogen, fibronectin, plasminogen, and vWF) are blocked combinatorically. The depletion efficiency of MRSA (Gram‐positive) (G) and carbapenem‐resistant *Escherichia coli* (CR *E. coli*, Gram‐negative) (H) (*n* = 3). Values are presented as the mean ± S.E.M. Statistical significance was calculated by a two‐tailed Student's *t*‐test. *p < 0.05; **p < 0.01; ***p < 0.001; NS, not significant.

We aimed to create a surface coated with diverse plasma proteins, enabling direct capture of a range of pathogens, encompassing both Gram‐negative and Gram‐positive bacteria (**Figure** 1A–C). Gram‐negative bacteria possess bacterial pili, which is essential for interacting with receptors on the host‐cell surface. In contrast, Gram‐positive bacteria utilize a family of proteins known as microbial surface components recognizing adhesive matrix molecules (MSCRAMMs) to initiate attachment to host tissues, thereby aiding in the establishment of infection.^[^ ^17^ ^]^ These bacterial adhesins engage with various components of the extracellular matrix (ECM), including fibrinogen, fibronectin, collagen, laminin, and vitronectin.^[^ ^18^, ^19^, ^20^ ^]^ Certain bacterial adhesins play a role in virulence strategies by interacting with host hemostatic factors such as fibrinogen, plasminogen, and vWF to facilitate infection.^[^ ^21^ ^]^ In plasma, specific ECM proteins and hemostatic factor proteins are present. Even after the formation of thrombi through plasma coagulation, ECM proteins, and hemostatic factor proteins persist abundantly on the surface of the thrombi. These proteins could act as a collective set of bacterial adhesin receptors, capable of capturing a diverse range of bacteria suspended in infected blood.

We quantitatively analyzed the plasma proteins found on the surface of the thrombi constructed in a Y‐shaped microfluidic device (Figure S1, Supporting Information). We validated the presence of plasma proteins, including fibrinogen, fibronectin, plasminogen, and vWF, on the surface of the cell‐depleted thrombi (CDT) through immunofluorescence staining (Figure 1D). As predicted, the CDT surpasses whole blood thrombi (WBT) in bacterial adhesin receptors’ density due to the presence of platelets and blood cell components in WBT, which diminish the exposure of bacterial adhesin receptors, such as fibrinogen and fibronectin, of thrombi (Figure 1D–F). Among the bacterial adhesin receptors in the CDT, fibrinogen fibers were the most abundant, followed by fibronectin, plasminogen, and vWF, of which the distribution aligns with the proportion of plasma proteins.^[^ ^22^ ^]^ vWF was observed to be the least abundant, likely due to its initial deposition on the surface occurring under shear flow conditions.^[^ ^23^, ^24^ ^]^

To quantitatively assess the contribution of each bacterial adhesin receptor on the CDT in capturing bacteria, we performed a combinatorial analysis, which involved blocking each adhesin receptor individually with its corresponding antibody. Fibrinogen and fibronectin emerged as key players in capturing both Gram‐positive (Figure 1G) and Gram‐negative bacteria (Figure 1H). This is likely attributed to their abundance on the surface of the CDT, facilitating the effective binding of bacteria.

### 2.2 Experimental Validation of Bacterial Margination toward Channel Surfaces

**Figure 2:**
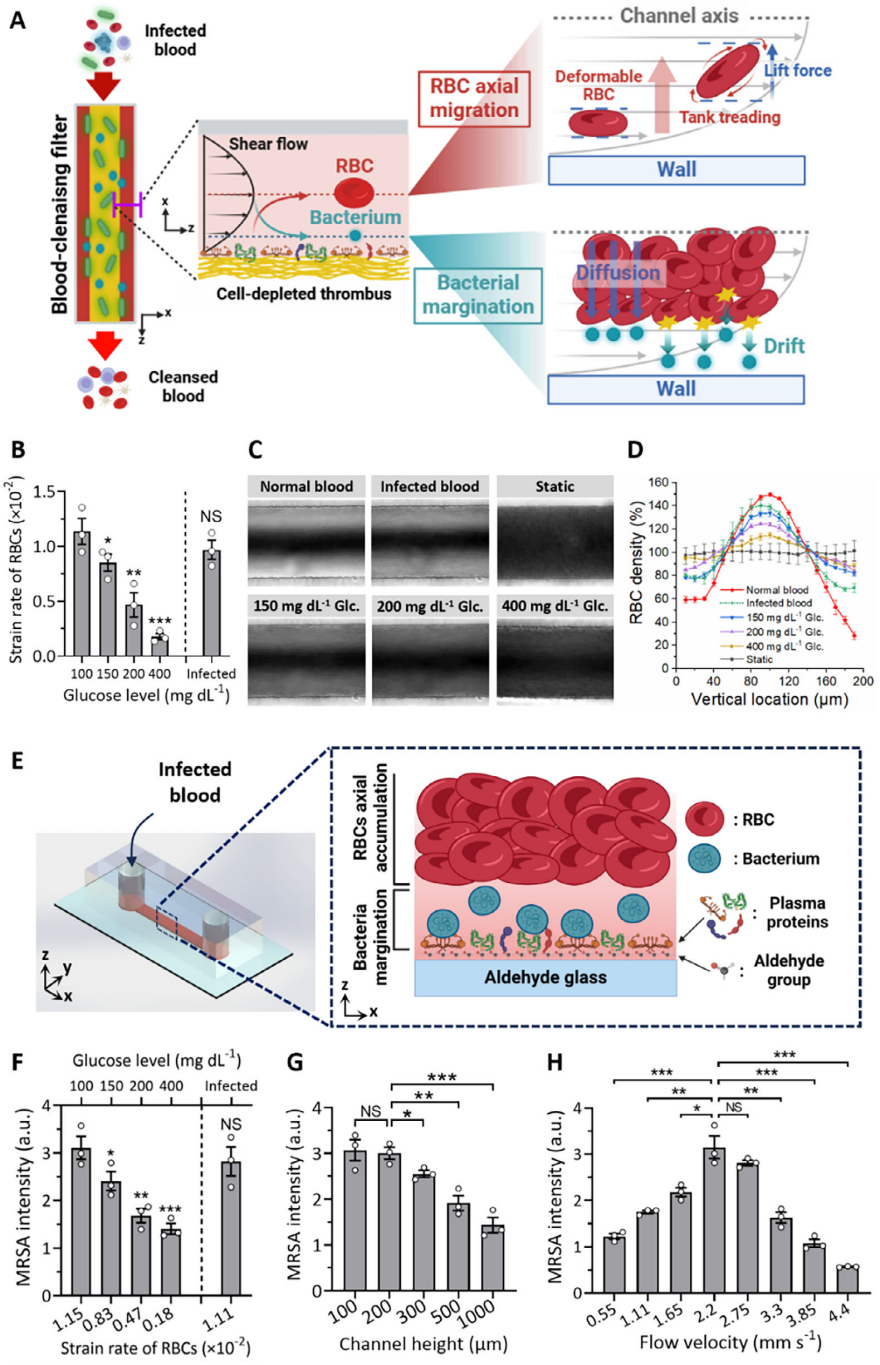
Axial accumulation of deformable RBCs induces bacterial margination, facilitating adhesion to the CDT surface. A) Axial migration of RBCs induces bacterial margination, facilitating bacterial adhesion to the CDT surface within the fluidic channel of the extracorporeal blood‐cleansing filter. As an RBC flows in shear flow, its membrane undergoes a tank‐treading motion, resulting in a steady‐state inclination of the cell relative to the flow direction. This orientation exposes the RBC to higher pressure differences along the cell in the shear gradient, generating an enhanced lift force directed toward the channel axis. In addition to the increased diffusion of bacteria due to their coexistence with axially accumulated RBCs, bacteria experience an additional drift migration caused by direct collisions with the axially accumulated RBCs. B) The strain rates of RBCs representing their deformability decrease significantly with the addition of glucose to whole blood. Although severe infection also reduces RBC deformability, the extent of this change is not statistically significant, even with infection in rats induced by a lethal dose of bacteria. Rat blood samples with varying glucose levels (150, 200, and 400 mg dL^−1^) and bacteremic blood of rats infected with a lethal dose of 10^5^ CFU mL^−1^ were used. C) Microscopic images demonstrate deformability‐induced axial migration of RBCs when flowing through a 200 µm‐high microfluidic channel. The axial migration of RBCs decreased significantly as the blood glucose level increased. D) Quantitative analysis of axial migration of RBCs shown in (C) with varying glucose concentrations and bacteremic blood (*n* = 3). E) A microfluidic device designed to evaluate bacterial adhesion depending on RBC deformability. CDT is formed on the aldehyde‐functionalized glass substrate. F–H) Quantitative assessment of bacterial adhesion to the CDT surface with varying conditions of the RBC deformability (strain rates) F), channel height G), and flow velocity H) (*n* = 3). Values are presented as the mean ± S.E.M. Statistical significance was calculated by a two‐tailed Student's t‐test. *p < 0.05; **p < 0.01; ***p < 0.001; NS, not significant.

It is crucial that bacteria in the bloodstream, flowing through an extracorporeal hemoadsorption device, are directed toward the surface where universal bacteria‐binding molecules can capture them. This is particularly important due to their inherently low diffusion coefficients (sizes above 1 µm in diameter), which hinder frequent interactions with solid matrices.^[^ ^25^ ^]^ In blood flow, RBCs migrate axially toward the center of confined channels, driven by their deformability and tank‐treading motion of cells.^[^ ^26^, ^27^ ^]^ This migration creates a cell‐free layer near the channel walls, where smaller, less deformable bacteria are displaced (**Figure** **2**A).^[^ ^28^ ^]^ Bacterial diffusivity in blood flow (3.41  ×  10^−11^ m^2^ s^−1^) is substantially higher than in the absence of RBC interactions (3.66  × 10^−13^ m^2^ s^−1^), promoting enhanced lateral migration toward the wall.^[^ ^29^, ^30^, ^31^, ^32^ ^]^ More detailed descriptions can be found in the Experimental Section.

Given that bacterial margination is driven by the axial migration of RBCs, it is essential to determine the minimum deformability of RBCs that enables this migration because their rigidity varies across different disease conditions, including diabetes, infection, and cancer.^[^ ^33^, ^34^, ^35^ ^]^ To identify the critical deformability of RBCs, we supplemented the normal whole blood of rats (≈100 mg dL^−1^ of glucose) with additional glucose at various concentrations (150, 200, and 400 mg dL^−1^) to demonstrate the reduced deformability of RBCs.^[^ ^36^ ^]^ The shear strains of the RBCs representing the deformability were assessed by squeezing them in a microfluidic channel with a central constriction (Figure S2, Supporting Information) and compared to those of RBCs from animal models with severe bacteremic infection. The deformability of RBCs significantly decreased as the concentration of glucose increased (Figure 2B–D). We determined the critical deformability of RBCs that facilitates axial migration (0.83 × 10^−2^), while severe infection reduces the strain rate of RBCs to a level ((0.98 ± 0.09) × 10^−2^), which does not compromise their movement of RBCs toward the center of the streamlines. These results support that bacterial margination, induced by axial migration of RBCs, can still be effective even in the treatment of individuals with severe infections.

We then assessed bacterial adhesion in whole blood while flowing through the microfluidic channel (Figure 2E; Figure S3, Supporting Information) to validate the key physical parameters mainly affecting bacterial capture on CDT surfaces.^[^ ^37^, ^38^ ^]^ Moreover, as anticipated from the RBC deformability data shown in Figure 2B–D, significantly fewer bacteria were captured on the CDT surfaces as RBC rigidity increased. However, the bacteria capture efficiency in RBCs from infected blood was similar to that in healthy blood (Figure 2F; Figure S4, Supporting Information) due to the negligible changes in deformability. Moreover, bacterial capture efficiency declines as channel heights increase, likely due to reduced fluidic shear rates within the microfluidic channel, which in turn diminish lift force acting on RBCs, as shown in Figure 2A. While channel heights of 200 µm or less maintained similar bacterial capture efficiency, the flow throughput is greater at a channel height of 200 µm. Therefore, we determined a channel height of 200 µm for our experimental design, achieving a balance between bacterial capturing capability and throughput (Figure 2G; Figure S5, Supporting Information).^[^ ^39^, ^40^ ^]^

We also identified an optimal flow rate of bacteremic blood for maximizing the capture efficiency because it is influenced by two opposing effects regarding flow velocity. As RBCs move at higher flow rates in blood, their axial migration is enhanced,^[^ ^41^ ^]^ leading to increased bacterial capture efficiency due to intensified collisions between bacteria and RBCs. However, it also results in an increased Stoke's drag force acting on the bacteria, which detaches them from the surface. To experimentally validate our hypothesis, we measured the bacteria capture efficiency on the CDT surfaces within the microfluidic device. As predicted, we observed a gradual increase in the number of captured bacteria with increasing flow rates, peaking at 2.2 mm s^−1^. Beyond this velocity, however, bacteria began to detach from the surface due to shear‐driven drag force (Figure 2H; Figure S6, Supporting Information). Based on these findings, we identified a flow velocity of 2.2 mm s^−1^ as the optimal condition, representing a balance between margination‐enhanced bacterial capture and their detachment induced by shear drag force (Note S1, Supporting Information).

### 2.3 Design and Fabrication of CDT Embedded in Helical Blades

**Figure 3:**
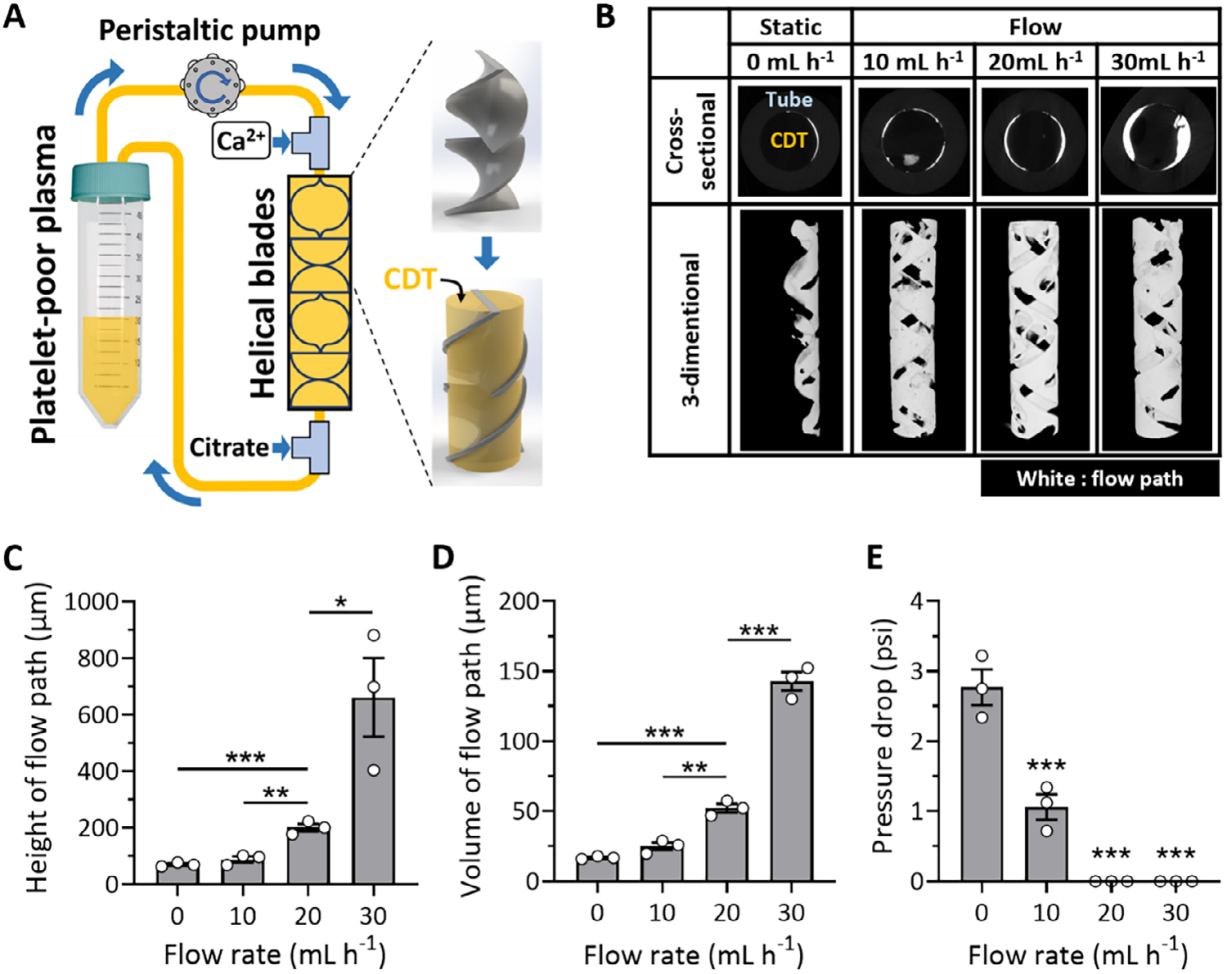
Fabrication and characterization of extracorporeal CDT filter (eCDTF). A) A schematic illustration demonstrating the fabrication process of eCDTF, depicting the progressive incorporation of CDT within the helical blades through continuous blending of platelet‐poor plasma and ionized calcium. B) Computed tomographic (CT) images displaying the established flow path (white) resulting from the flow‐driven fabrication process under varying flow rate conditions (0, 10, 20, and 30 mL h^−1^). C–E) Quantitative analysis of the flow path's height C), volume D), and pressure drop E) at various flow rates during the eCDTF fabrication process (0, 10, 20, and 30 mL h^−1^) (*n* = 3). The height of flow paths must remain under 300 µm to facilitate RBC axial migration and bacterial margination while minimizing pressure drop within this range of flow rates. Values are presented as the mean ± S.E.M. Statistical significance was calculated by a two‐tailed Student's *t*‐test. *p < 0.05; **p < 0.01; ***p < 0.001; NS, not significant.

Combining all parameters and material conditions we examined above, we designed an extracorporeal blood cleansing device that removes a wide range of bacteria from whole blood. The fluidic geometry must maximize the surface area per unit volume while maintaining the ability to flow blood at a rate greater than > 10 mL h^−1^ for treating at least small animals using a single device module and effectively mixing blood components, given the low Peclet number at the microscale. We employed multiple helical blade components inserted in medical tubing because they can serve as stable anchors for CDT while circulating blood through the extracorporeal device and facilitate continuous mixing of platelet‐poor plasma with ionized calcium when constructing CDT within the helical blade components. Moreover, these helical structures also enhance microbial attachment to the CDT surface, as predicted by particle tracing simulations using the COMSOL Multiphysics software (Movies S1 and S2, Supporting Information). Platelet‐poor plasma was continuously infused with a calcium chloride solution and flowed through the helical blade‐containing medical tubing, effectively forming CDT trapped in every segment of the helical blades (**Figure** **3**A). Flow paths generated within the device were visualized and assessed by 3D computed tomography (CT) scanning (Figure 3B). The heights of the flow paths increased as the flow rates of the infused platelet‐poor plasma solution rose. This is attributed to the flow path being predisposed to a wider cross‐sectional area to minimize pressure drop across the channel at elevated flow rates. At flow rates of 30 mL h^−1^ and above, the flow path height formed between the helical blades and the tubing surface was 661 ± 139 µm (Figure 3C), resulting in the largest volume of the flow path (Figure 3D). However, at this height, RBC axial migration becomes ineffective, leading to reduced bacterial capture efficiency on the surface. While flow rates below 20 mL h^−1^ allow for flow path heights less than 300 µm (Figure 3C), these rates were not suitable due to a significant pressure drop when flowing blood through the device (Figure 3E). Therefore, we determined 20 mL h^−1^ as the optimal flow rate for fabricating the extracorporeal CDT filter (eCDTF), as it was predicted to facilitate effective bacterial margination while minimizing pressure drop.

### 2.4 Blood Components Affecting Thrombi‐Based Extracorporeal Bacterial Depletion

**Figure 4:**
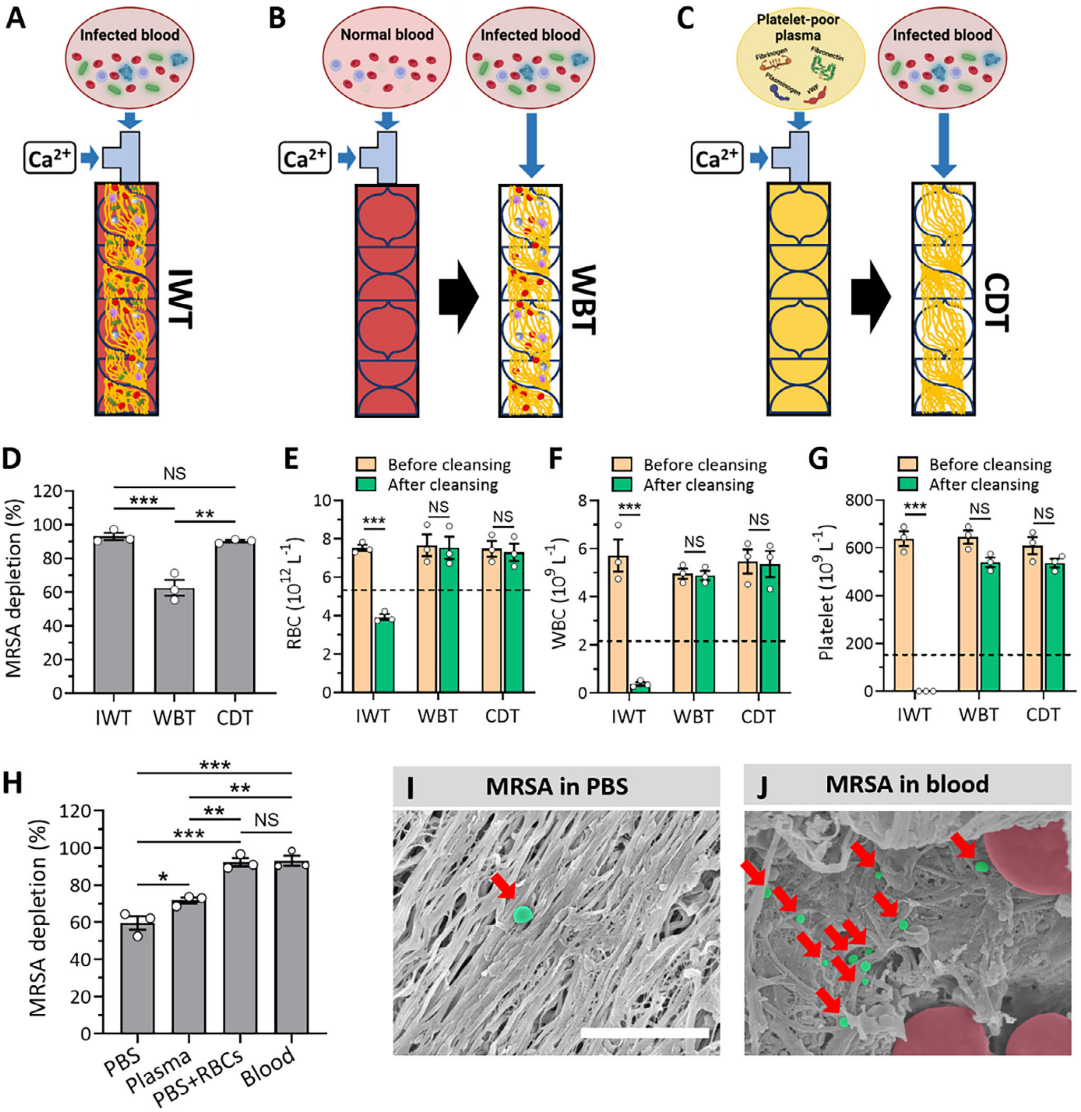
Characterization of several types of thrombi‐based bacterial hemoadsorption and the role of opsonins in bacterial capturing efficiency. A–C) Illustrations of hemoadsorption processes utilizing thrombi from blood with different conditions. The hemoadsorption filters constructed with bacteria‐infected whole blood thrombi (IWT) (A) occurring concurrently as infected whole blood passes through the helical blades in the tubing, preformed whole blood thrombi (WBT) B), and preformed cell‐depleted thrombi (CDT) C). D) A comparative analysis of MRSA depletion efficiencies using IWT, preformed WBT, and preformed CDT. E‐G) Blood cell counts, including RBCs E), WBCs F), and platelets G), measured after hemoadsorption with IWT, preformed WBT, and preformed CDT. H) MRSA depletion rates (%) indicate that bacterial depletion by the preformed CDT in the device is primarily influenced by RBC axial migration rather than the presence of opsonins. I,J) Scanning electron microscopy (SEM) images of MRSA adhered to the CDT surfaces after bacteria‐containing PBS I) and whole blood J) pass through the preformed CDT. Red arrows indicate MRSA pseudo‐colored in green. Scale bar: 5 µm. Values are presented as the mean ± S.E.M. Statistical significance was calculated by a two‐tailed Student's *t*‐test. *p < 0.05; **p < 0.01; ***p < 0.001; NS, not significant.

Several types of thrombi can be formed when incorporated into the helical blades in the tubing, including thrombi from infected or healthy whole blood, as well as platelet‐poor plasma. Specifically, bacteremic blood itself tends to form blood clots simultaneously (infected whole blood thrombi, IWT) while exposed to shear stress, as infection can induce thrombosis (**Figure** **4**A).^[^ ^42^ ^]^ Alternatively, thrombi structures can be prefabricated in the helical blades using normal whole blood (whole blood thrombi, WBT) before flowing bacteremic blood (Figure 4B). These two thrombi fabrication approaches mimic the conditions encountered when infected individuals are treated with conventional extracorporeal devices, where infected blood may either directly coagulate during passage through the device or come into contact with pre‐existing whole blood thrombi already trapped within the devices. We compared the characteristics of CDT (Figure 4C) with those two experimental conditions (IWT and WBT) to validate the superiority of CDT constructed in the device and assess the potential adverse effects of IWT and WBT for extracorporeal bacterial depletion using methicillin‐resistant *Staphylococcus aureus* (MRSA) in blood.

MRSA (10^5^ CFU mL^−1^) depletion in whole blood (20 mL) flowing through IWT and CDT resulted in 93.00 ± 2.14% and 90.28 ± 0.65%, respectively, while WBT hemoadsorption removed only 62.60 ± 4.64% of MRSA in the blood (Figure 4D). The compromised bacterial depletion efficiency of WBT is attributed to blood components of whole blood, such as RBCs and platelets, blocking adhesin receptor proteins on the surface of thrombi, which disrupt bacterial capture. Moreover, in IWT hemoadsorption, a significant number of critical blood components, including RBCs, white blood cells (WBCs), and platelets, were also reduced, most likely due to simultaneous blood coagulation of infected blood during the passage (Figure 4E–G). However, the CDT hemoadsorption largely preserved most blood components while maintaining high bacterial depletion efficiency, demonstrating the superiority of eCDTF over other types of thrombi‐based hemoadsorption approaches (Figure 4D–G).

We also investigated the extent of the role of opsonins in capturing bacteria using eCDTF. While the depletion rate of MRSA spiked in phosphate‐buffered saline (PBS) was 59.63 ± 3.65% (Figure 4H,I), it increased to 71.75 ± 1.61% (*p* < 0.05) in platelet‐poor plasma with various opsonin molecules (Figure 4H). However, MRSA in PBS supplemented only with RBCs exhibited a significantly high depletion rate of 92.29 ± 2.23%, comparable to that in whole blood (93.14 ± 2.69%) (*p* = 0.41) (Figure 4H,J). This indicates that while opsonins may enhance bacterial capture to some extent, the bacteria depletion using eCDTF may not strictly depend on their presence once bacteria migrate toward the CDT surface. Rather, bacterial margination driven by RBC axial migration appears to play a more critical role in capturing bacteria within eCDTF.

### 2.5 Removal of Various Bacteria from Whole Blood Using an Extracorporeal CDT Filter In Vitro

**Figure 5:**
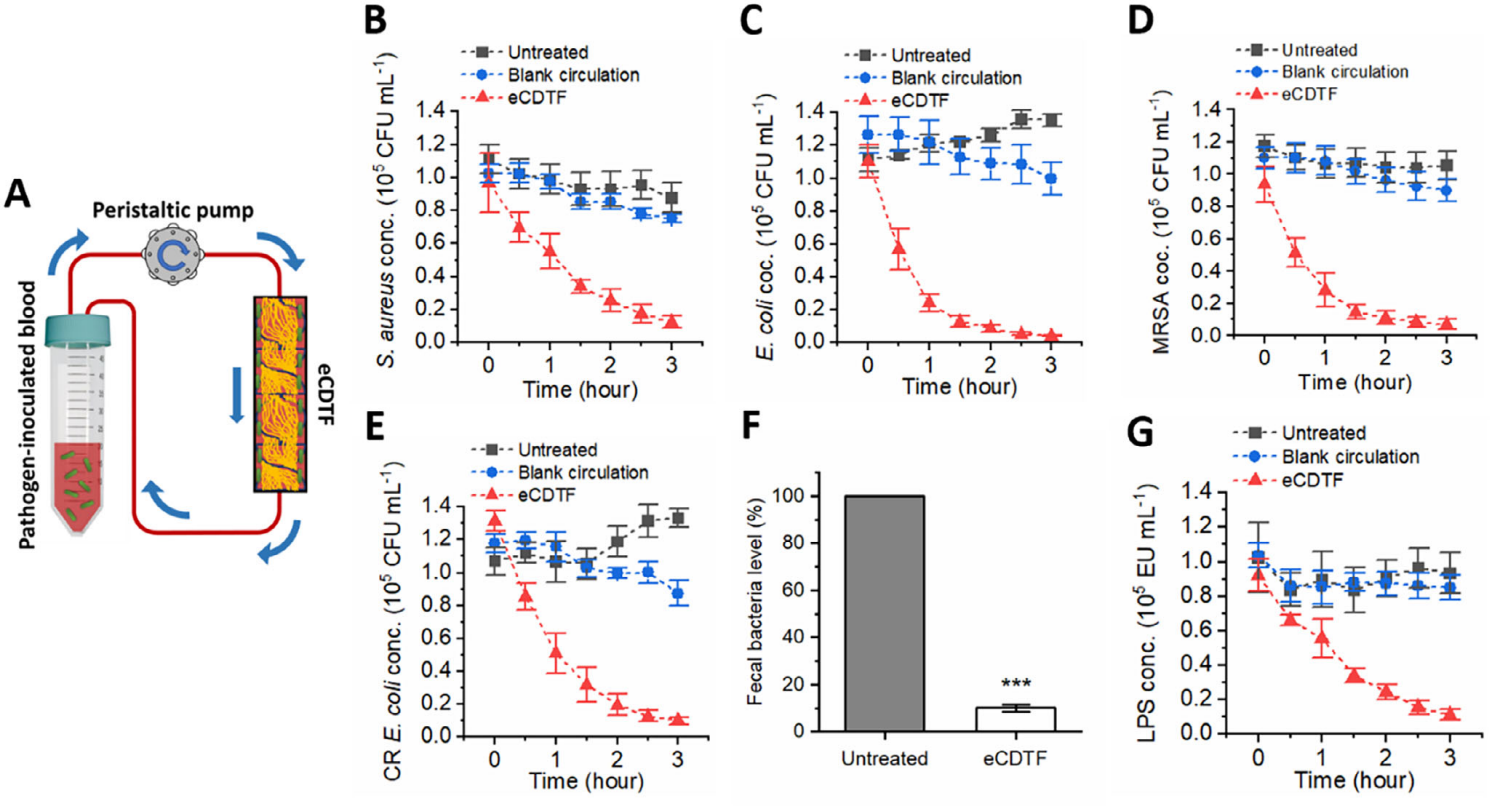
Continuous removal of various bacteria from whole blood using eCDTF in vitro. A) A schematic illustration of the eCDTF circulating bacteria‐infected blood at 20 mL h^−1^ for assessing the depletion efficiency. B,C) Quantification of the bacteria removal efficiency for Gram‐positive *S. aureus* B) and Gram‐negative *E. coli* C) using eCDTF compared to untreated or blank circulation without preformed CDT on helical blades of the device (*n* = 3). D,E) The quantitative bacterial removal efficiency of antibiotic‐resistant strains: MRSA (D) and CR *E. coli* E) during the blood circulating through eCDTF (*n* = 3). F) More than 90% of human fecal microbes spiked in whole blood were depleted after treating the blood with eCDTF for 3 h (*n* = 3). G) Continuous removal of endotoxin (LPS) from whole blood using eCDTF for 3 h. Values are presented as the mean ± S.E.M. Statistical significance was calculated by a two‐tailed Student's *t*‐test. *p < 0.05; **p < 0.01; ***p < 0.001; NS, not significant.

We quantified the bacteria depletion efficiency and diversity of bacteria that our device can remove from whole blood (**Figure** **5**A). The eCDTF successfully eliminated ≈90% of major pathogenic bacteria species, including *Staphylococcus aureus* (*S. aureus*), *Escherichia coli* (*E. coli*), MRSA, and carbapenem‐resistant *E. coli* (CR *E. coli*), within 3 h post‐treatment in vitro (Figure 5B–E). Despite the acquisition of antimicrobial resistance affecting outer membrane components, such as penicillin‐binding protein 2a (PBP2a) in MRSA and outer membrane porins in CR *E. coli*, the eCDTF retained its efficacy in capturing diverse bacterial strains. This generic binding capability is attributed to the presence of multiple types of MSCRAMMs in *S. aureus* and fimbrial adhesins in the pilus structure of *E. coli*, facilitating the binding of bacterial cells to the CDT surface. To validate the extended capability to capture an even broader range of bacteria, human fecal microbes spiked in human whole blood were passed through eCDTF, mimicking bacteremia caused by fecal peritonitis.^[^ ^43^, ^44^ ^]^ We identified 76 bacterial species within the fecal microbiome using next‐generation sequencing (NGS). The eCDTF hemoadsorption exhibited over 90% depletion of fecal microbes from human whole blood. Among the bacterial species, 37 were completely eradicated, while 23 species exhibited a removal efficiency ranging from 90% to 99.99% (Figure 5F; Table S1, Supporting Information). Furthermore, the eCDTF hemoadsorption successfully removed 87.20% of 10^5^ endotoxin units mL^−1^ of LPS within 3 h (Figure 5G). This effective capture is attributed to the electrostatic interaction between lysine and arginine residues on the CDT surface and the negatively charged phosphates of the phospho‐glucosamine residues in the lipid A backbone of LPS.^[^ ^45^ ^]^ Moreover, due to the size of LPS smaller than bacterial cells (< µm), they facilitate more frequent collisions with the CDT surface, potentially resulting in greater depletion efficiency compared to that of bacteria.^[^ ^29^, ^46^ ^]^

### 2.6 Extracorporeal Blood‐Cleansing Treatment of Bacteremia Models in Rats

**Figure 6:**
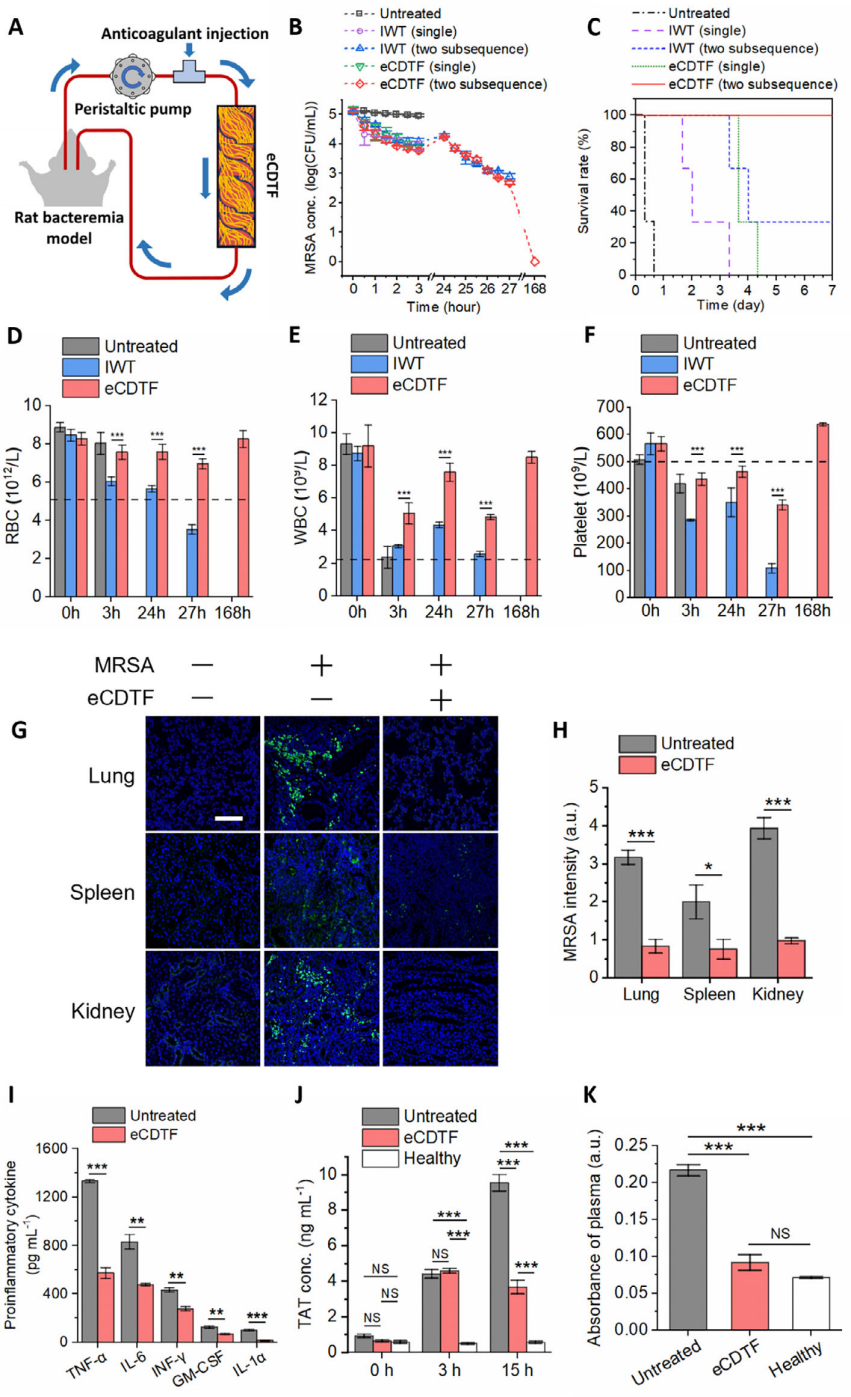
In vivo extracorporeal blood‐cleansing treatment in MRSA‐infected rats and subsequent restoration of hematological parameters and immune homeostasis. A) Schematic of an experimental setup of an eCDTF for extracorporeally treating bacteremia rats lethally infected with MRSA. B) Bacterial levels in peripheral blood of the MRSA‐infected rats during the 3‐h extracorporeal blood treatment (either a single round or two‐subsequent rounds), compared to those treated with infected whole thrombus (IWT) concurrently formed in the helical blades within the tubing, as well as an untreated control group (*n* = 3). C) Kaplan–Meier curves depicting increased survival rates in the MRSA‐infected rats when the 3‐h treated rats were subsequently treated again next day using eCDTF (two subsequence – red solid, single – green dotted), compared to IWT treatment (two subsequence – blue dashed, single – purple dashed) and an untreated control group (black dash‐dotted) (*n* = 3). D–F) Complete blood count (CBC) outcomes, including RBC D), WBC E), and platelet F) counts, indicating hematological parameter restoration post‐eCDTF treatment; significant blood component loss followed IWT treatment. G) Immunofluorescence microscopy images showing the lung, spleen, and kidney tissues harvested from healthy (1^st^ row), untreated (2^nd^ row), and MRSA‐infected rats treated with eCDTF (3^rd^ row, two subsequent rounds) (MRSA, green; cell nucleus, blue). Scale bar: 100 µm. H) Quantitative measurement of MRSA levels in the major organs using the immunofluorescence images across six random fields (*n* = 3). I) Notable reduction in proinflammatory cytokines—tumor necrosis factor‐α (TNF‐α), interleukin‐6 (IL‐6), interferon‐γ (INF‐γ), granulocyte‐macrophage colony‐stimulating factor (GM‐CSF), and IL‐1α—in the blood of the MRSA‐infected rats following eCDTF treatment, compared to an untreated control group (*n* = 3). J) Analysis of thrombin‐antithrombin (TAT) complex concentrations in the blood of MRSA‐infected rats shows that lower TAT levels were maintained, while those levels increased in the untreated groups at 15 h post‐single round of eCDTF treatment (*n* = 3). K) Hemolysis analysis via platelet‐poor plasma absorbance measurement at 540 nm indicates minimal hemolysis in the blood of MRSA‐infected rats when treated with eCDTF, whereas significant hemolysis was observed in the untreated rat blood (*n* = 3). Values are presented as the mean ± S.E.M. Statistical significance was calculated by a two‐tailed Student's *t*‐test. *p < 0.05; **p < 0.01; ***p < 0.001; NS, not significant.

We then validated the preclinical utility of eCDTF in bacteremia models in rats lethally infected with MRSA (**Figure** **6**A). The rats with bacteremia underwent extracorporeal treatment with eCDTF for 3 h and exhibited a gradual decrease of MRSA levels in the bloodstream by 94.25%. In contrast, the control group without treatment showed consistently elevated MRSA levels, ≈10^5^ CFU mL^−1^, over the entire 3‐h observation period (Figure 6B). The MRSA levels in the bloodstream surged within the next 24 h, potentially due to their multiplication within the bloodstream and reflux from other organs back into the bloodstream.^[^ ^47^, ^48^ ^]^ Following that, we implemented an additional 3‐h blood‐cleansing therapy for the rats on the subsequent day. This led to a gradual decrease in MRSA levels in the bloodstream once more, achieving a total reduction of 99.08% in MRSA levels. After 168 h of survival, the MRSA concentration in the blood dropped to levels undetectable with PCR (< 10^2^ CFU mL^−1^).^[^ ^49^, ^50^ ^]^ This suggests that serial hemoadsorption treatments using our eCDTF reduced pathogen levels that became manageable by the rat's immune system (Figure 6B).

Furthermore, we explored the potential adverse effects of bacteria depletion achieved by IWT hemoadsorption (Figure 4A) in bacteremia in rats. This experimental condition mimics a clinical scenario where bacteremic patients undergo treatment with various types of extracorporeal devices, occasionally leading to the progressive formation of blood clots akin to IWT while blood continues to flow through them.^[^ ^51^, ^52^, ^53^ ^]^ Despite bacterial reduction in the blood in IWT‐treated rats comparable to other eCDTF‐treated groups, they failed to survive for the seven‐day observation period even after two subsequent treatments (Figure 6C). These outcomes are attributed to compromised hematological parameters resulting from progressive coagulation in IWT during the extracorporeal blood‐cleansing treatment (Figure 6D‐F). However, eCDTF treatment preserved blood component levels, including RBCs, WBCs, and platelets, while significantly reducing bacterial levels both in the bloodstream and the major organs such as the lungs, spleens, and kidneys of the infected rats (Figure 6G,H), consistent with observations from previous studies.^[^ ^47^, ^54^, ^55^ ^]^ Furthermore, a notable decrease in proinflammatory cytokine levels in the bloodstream, including tumor necrosis factor‐α (TNF‐α), interleukin‐6 (IL‐6), interferon‐γ (INF‐γ), granulocyte‐macrophage colony‐stimulating factor (GM‐CSF), and IL‐1α, was observed after a 7‐day survival period following two subsequent rounds of eCDTF treatments (Figure 6I). Lowering pathogens levels in both the bloodstream and major organs is likely responsible for the decline of proinflammatory activities.

The concentration of thrombin‐antithrombin complex (TAT), an indicator of net activation of coagulation, exhibited a slight increase immediately after a single 3‐h eCDTF treatment. This elevation was likely triggered by the extracorporeal blood circulation, which is known to activate coagulation in the blood.^[^ ^56^, ^57^ ^]^ Nevertheless, due to the decrease in pathogen loads resulting from two subsequent rounds of eCDTF treatments, the TAT levels remained below 4 ng mL^−1^ for 15 h in the eCDTF‐treated rats (Figure 6J). However, in the untreated rats, TAT levels increased up to 10 ng mL^−1^ within 15 h, potentially leading to disseminated intravascular coagulation (DIC).^[^ ^58^ ^]^ Furthermore, the eCDTF‐treated animals had minimal hemolysis levels, whereas the untreated rats exhibited significant hemolysis (Figure 6K). This difference was attributed to the combined therapeutic effects of reduced pathogen loads and restored systemic inflammation in the infected animals because infection triggers the release of hemoglobin from RBCs.^[^ ^59^ ^]^

## 3 Discussion

Numerous extracorporeal therapeutic approaches have been investigated to eliminate the source of an infection that persistently triggers a fatal inflammatory response. Recently, a variety of commercially available hemofilters utilizing surface‐adsorption‐based techniques have been introduced in the clinical setting.^[^ ^60^, ^61^ ^]^ However, hemofilters coated with a specific functional reagent are designed to target the removal of endotoxins or debris rather than intact bacteria. While conventional hemoadsorption devices lack a specific strategy for bacterial removal, sporadic clinical studies have reported a reduction in bacterial loads in the bloodstream.^[^ ^15^, ^16^ ^]^ Nevertheless, no clear explanations were provided for those inconsistent clinical outcomes. These challenges of traditional hemoadsorption devices are attributed to the lack of a strategy for delivering intact bacteria to the functionalized surface and universal bacteria‐capturing receptors present on the surface of blood‐filtering devices, which are not compromised even after multiple passages of whole blood. In this study, we developed an extracorporeal device constructed with universal bacteria‐binding CDT, maintaining efficient bacteria‐capturing capabilities even after multiple passages of whole blood. Furthermore, we theoretically designed and fabricated the blood flow paths in the eCDTF that facilitate the effective margination of bacteria toward the CDT surface, where they can be efficiently captured. Our demonstration also suggests avoiding progressive blood clot formation in extracorporeal devices and even in the host body in a clinical setting because it can compromise major hematological parameters, leading to increased lethality.

The combination of bacterial adhesin receptors originating from platelet‐poor plasma, such as fibrinogen, fibronectin, plasminogen, and vWF, interacts with multiple bacterial adhesins on microbial surfaces and endotoxins. The multifunctional properties of CDT enable the elimination of Gram‐positive, Gram‐negative, and antibiotic‐resistant bacteria, while the previous hemoadsorption methods eliminate only Gram‐negative bacteria along with concomitant antibiotic treatment.^[^ ^11^, ^12^, ^13^ ^]^ The application of this capability we demonstrated could prove invaluable in addressing the challenge of blood culture‐negative bacteria removal, as conventional broad‐spectrum antibiotics can often impose significant burdens on patients when treating unculturable bacteria.^[^ ^62^ ^]^

For clinical applications, several factors need to be considered to enhance therapeutic efficacy. Although our results demonstrated that even severe infection did not significantly increase the RBC stiffness (Figure 2B), a few other medical conditions that decrease the strain rate of RBCs below 0.83 × 10^−2^, such as sickle cell disease, chronic inflammatory diseases, and aging,^[^ ^63^, ^64^, ^65^ ^]^ could potentially result in diminished bacterial margination and depletion in eCDTF treatment. Consequently, adjustments in the treatment protocol may be necessary, such as RBC transfusion, which can supplement deformable RBCs, re‐inducing bacterial margination to the surface again. To scale up the device for human clinical trials, we can simply parallelize eCDTF to increase flow rates while maintaining bacterial depletion efficiency (Figure S7, Supporting Information). The use of human plasma for constructing an extracorporeal hemoadsorption device offers advantages, as blood plasma transfusion has been widely regulated for safety and quality control. The cost of plasma required for device fabrication would also be minimal, given the market price of human plasma.^[^ ^66^, ^67^ ^]^ Thus, considering the availability of human plasma and the reproducibility and simplicity of device fabrication, the production of eCDTF for clinical use is expected to be efficient once we comply with ISO regulations.

## 4 Experimental Section

### Blood Collection and Preparation

Blood collection procedures for both animals and humans adhered to protocols approved by the Institutional Animal Care and Use Committee (IACUC) and the Institutional Review Board (IRB) at Ulsan National Institute of Science and Technology (UNIST). Following IRB approval (UNISTIRB‐19‐23‐C), human blood samples were sourced from the Korea Red Cross. Rat blood samples were collected post‐IACUC approval (UNISTIACUC‐22‐70). For the preparation of hyperglycemic blood, 50–300 mg dL^−1^ of glucose was added to whole blood.

### Platelet‐Poor Plasma Preparation

Following IRB approval (UNISTIRB‐19‐23‐C), human platelet‐poor plasma was prepared by thawing fresh frozen plasma (FFP) obtained from the Korea Red Cross. After receiving IACUC approval (UNISTIACUC‐22‐70), rat whole blood was collected for rat platelet‐poor plasma preparation. The blood was centrifuged at 800 × *g* for 15 min. Subsequently, the plasma layer was carefully extracted, avoiding the buffy coat and red blood cell layers, to minimize platelet contamination. The resulting platelet‐poor plasma was subjected to quality control measures to ensure platelet counts were below 10 000 platelets µL^−1^, consistent with recommended thresholds.

### A Theoretical Model for RBCs Axial Migration and Bacterial Margination

In blood flow, RBCs confer non‐Newtonian characteristics to the flow due to their high‐volume concentration and deformable nature.^[^ ^32^, ^68^ ^]^ A prominent characteristic of blood flow is the axial accumulation of RBCs in confined channels, a phenomenon distinct from the behavior of spherical rigid particles.^[^ ^69^, ^70^ ^]^ As deformable RBCs undergo tank‐treading, they achieve a steady‐state inclination relative to the flow direction.^[^ ^26^, ^27^ ^]^ This orientation results from a balance between the resistive moment generated by the tank‐treading motion of the cell membrane and the moment induced by the vorticity of the shear flow.^[^ ^71^, ^72^ ^]^ The asymmetric shape and orientation of RBCs in shear flow create uneven fluid velocities around the cells, generating an enhanced hydrodynamic lift force toward the channel axis (Figure 2A). This lift force enables RBCs to migrate away from the low‐velocity regions (high pressure) near the channel wall toward the center, where the flow velocity is higher (low pressure).^[^ ^73^, ^74^, ^75^ ^]^ Axial accumulation of RBCs occurs prominently in narrow microfluidic channels (<≈300 µm), whereas in wider channels (>≈300 µm) with relatively lower shear rates, this phenomenon is diminished.^[^ ^40^, ^76^ ^]^ As a result, a cell‐free layer forms near the channel wall. Bacteria, being smaller and less deformable than RBCs, tend to be displaced from the central flow and migrate toward this outer cell‐free plasma layer due to interactions with axially accumulated RBCs.^[^ ^28^ ^]^

The Brownian diffusivity of bacteria in blood plasma, derived from Einstein's theory of Brownian motion, is ≈3.66  × 10^−13^ m^2^ s^−1^, as given by the equation^[^ ^29^ ^]^

(1) $$D_{\mathrm{Brownian}}=\frac{k_{B}T}{6\pi \eta r}$$

where *k_B_* is the Boltzmann constant, *T* is thermodynamic temperature, η is the dynamic viscosity of blood plasma, and *r* is the radius of the bacteria. Zydney and Colton (1988) proposed an effective diffusivity model for platelets in whole blood that incorporates shear rate ($\dot{\gamma }$) and hematocrit (φ_*RBC*_) and diameter of RBCs (*d_RBC_*)—a model still widely applied.^[^ ^30^, ^31^, ^32^ ^]^ Given that bacteria are similar in size to platelets and more rigid than RBCs, this diffusivity model was extended to describe bacterial diffusion in blood

(2) $$\begin{array}{ccc} D & = & 0.15\dot{\gamma }{\left(\frac{d_{\mathrm{RBC}}}{2}\right)}^{2}\varphi_{RBC}{\left(1-\varphi_{RBC}\right)}^{0.8}\\ & & +0.15\dot{\gamma }{\left(\frac{d_{\mathrm{bacteria}}}{2}\right)}^{2}\varphi_{\mathrm{bacteria}}+D_{\mathrm{Brownian}} \end{array}$$

The effective diffusivity of bacteria influenced by axially accumulated RBCs is ≈3.41  ×  10^−11^ m^2^ s^−1^. Consequently, when bacteria coexist with axially migrated RBCs in the bloodstream, their lateral dispersion toward the channel wall is significantly enhanced compared to when bacteria are suspended in plasma alone or in a flow with uniformly dispersed RBCs.

However, traditional diffusive equations for platelet transport fail to predict excess platelet concentrations near the channel wall. To address this, Eckstein and Belgacem developed a phenomenological drift‐diffusion model for platelet motion in blood flow, incorporating a drift term that accounts for the gradient of rheological potential ((ϕ), which derives drift migration of platelets.^[^ ^77^ ^]^ This drift migration of platelets can be applied to target bacteria that are similar in size to platelets and exhibit greater rigidity than RBCs. The flux of the bacteria is given by

(3) $$j=-\frac{\partial \phi }{\partial y}c-D\frac{\partial c}{\partial y}$$

where *c* is the bacteria concentration, and *D* is effective diffusivity. Due to the concentration gradient in the wall‐normal direction, variations in the collision frequency between bacteria and RBCs are expected to drive bacteria drift toward the wall (Figure 2A). The inclusion of a drift migration is also predicted to enhance the amount of bacteria deposited on the wall.^[^ ^77^ ^]^ Recent studies have further aligned computational simulations with theoretical models by incorporating a drift term that reflects the deformability of RBCs, narrowing the gap between theoretical predictions and observed behaviors in blood flow.^[^ ^78^, ^79^ ^]^

### RBC Deformability Assessment

Pressure‐driven flow experiments were conducted to evaluate RBC deformability in a microfluidic constriction, with deformation quantified using high‐speed imaging.^[^ ^80^, ^81^ ^]^ The microfluidic device, fabricated from polydimethylsiloxane (PDMS), featured a straight channel (width × height × length: 100 µm × 20 µm × 1.1 cm) with a single constriction (width × height × length: 40 µm × 20 µm × 100 µm). Rat RBCs were collected from a healthy donor and used within 2 h to minimize degradation of deformability.^[^ ^82^ ^]^ 6 µL of whole blood was diluted in 500 µL of phosphate‐buffered saline (PBS) and washed three times in PBS by centrifugation at 3000 rpm for 3 min at room temperature. The RBC suspension was then introduced into the microfluidic device at a flow rate of 1 µL min^−1^ using a syringe pump (Pump 11 Pico Plus Elite, Havard Apparatus, MA, USA).

RBC deformation during transit through the constricted channel was analyzed using high‐speed imaging. Videos were recorded at ≈3600 frames per second with a high‐speed camera (Phantom S991, Phantom, NJ, USA) mounted on a light microscope (Olympus CX23, Olympus, Tokyo, Japan) with a 40× objective. The maximum observed length (*L_max_*) and initial length (*L_in_*) of each RBC were determined. Nondimensional strain rate ($\overset{\prime }{Y}$) was calculated for each cell using the following equation:^[^ ^80^ ^]^

(4) $$\overset{\prime }{Y}=\frac{L_{max}-L_{in}}{L_{in}\dot{\gamma }\Delta t}$$

where *L_max_* − *L_in_* represents the change in RBC length, $\dot{\gamma }$ is the imposed shear rate, and Δ*t* is the time interval over which the deformation occurred.

### Assessment of Axial Accumulation of RBCs

Grayscale intensities along the cross‐sectional vertical line of blood flow within a 200 µm‐wide microfluidic channel were measured using ImageJ software (NIH, MD, USA). The RBC density (%) was determined based on the assumption that it is zero in plasma and 100% in the static condition of whole blood within a microfluidic channel. The differences between grayscale intensities at specific vertical locations (*I*) and the grayscale intensity of platelet‐poor plasma (*I_plasma_*) were compared with the difference between the grayscale intensity of static condition whole blood (*I_static_*) and *I_plasma_*. The equation of RBC density is given by

(5) $$\mathrm{RBC}\;\mathrm{density}\;\;\left(\%\right)=\;\frac{I_{\mathrm{plasma}}-\;I}{I_{\mathrm{plasma}}-I_{\mathrm{static}}}\times 100$$

### Device Fabrication for Investigating Bacterial Margination

The microfluidic device employed in this study was crafted through standard SU‐8 photolithography, utilizing a blend of PDMS precursor and a curing agent in a 10:1 weight ratio. This device showcases a straight channel (width × height × length: 1000 µm × 200 µm × 40 mm) with a distinct inlet and outlet. An aldehyde‐coated glass slide served as the substrate to optimize protein immobilization within the microchannel.

To ensure the PDMS substrate bonded to the aldehyde glass slide without harming the aldehyde‐coated layer within the potential microchannel, a Schiff‐base linkage was leveraged. This linkage formed between the aldehyde groups on the glass slide and the primary amine groups on the (3‐Aminopropyl)triethoxysilane (APTES) treated PDMS. The PDMS device surface, including the microchannel, was first activated using oxygen plasma. Subsequently, it was soaked in a 10% (v/v) APTES solution in anhydrous ethanol for an hour at ambient temperature. Following a nitrogen stream drying process, the PDMS was carefully aligned and pressed onto the aldehyde glass slide. Bonding via a condensation reaction was enhanced by incubating the integrated device for 5 h at 37 °C.

To functionalize the channel surface with adhesin receptor proteins, platelet‐poor plasma was introduced into the channel. The channel was then incubated with the platelet‐poor plasma for 1 h at room temperature before being rinsed with a 1×PBS solution. Subsequently, to mitigate nonspecific binding, the channel was saturated with a 2% bovine serum albumin (BSA) solution and allowed to incubate for another hour at room temperature.

### Device Fabrication for Observing Adhesin Receptor Proteins on the CDT Surface

In the initial step, a Y‐shaped pattern consisting of one straight channel (width × height × length: 500 µm × 500 µm × 2 cm) was micromachined onto a polymethylmethacrylate (PMMA) substrate using a computer numerical control (CNC) milling machine (David 3020C, David, Incheon, South Korea). A blend of PDMS precursor and curing agent, in a 10:1 mass ratio, was then cast onto this micromachined PMMA template. After the PDMS mixture was degassed to eliminate bubbles, it was cured at 60 °C for 2 h. The cured PDMS substrate was then carefully detached from the PMMA template. Next, a glass slide was aligned onto the PDMS substrate and was held in place through clamping. Platelet‐poor plasma was steadily introduced into one inlet at a rate of 2 mL h^−1^ over an hour, while 6.5 mg mL^−1^ of CaCl^2^ was simultaneously introduced into another inlet. Following the coagulation of the platelet‐poor plasma, the glass was removed to inspect the surface of CDT.

### Immunofluorescence Staining

Both the CDT surfaces and CDT‐coated aldehyde glass surface were first fixed using 10% formalin and subsequently blocked with a 2% BSA buffer for 1 h. Following a rinse with a 1×PBS solution, these samples underwent incubation with primary antibodies, suspended in a blocking buffer solution at 4 °C overnight. These primary antibodies were diluted in 2% BSA buffer, with the following ratios: anti‐fibrinogen, anti‐fibronectin, anti‐plasminogen, and anti‐von Willebrand Factor (all from Abcam) at a 1:100 dilution each. After another PBS wash, the samples were exposed to secondary antibodies: donkey anti‐sheep Alexa Fluor 594, goat anti‐rabbit Qdot 655, goat anti‐rabbit Alexa Fluor 405, and goat anti‐rabbit Alexa Fluor 488 (all from Thermo Fisher Scientific), each diluted at 1:100. This incubation took place at room temperature for 2 h. When MRSA was identified on the surface, it was labeled using anti‐*S. aureus* (Abcam) antibody conjugated with goat anti‐rabbit Alexa Fluor 488.

### CDT Construction in Helical Blades in Medical Tubing

Both static and flow‐conditioned plasma coagulation was performed in helical blade‐mounted tubing. The helical blade structure comprises 12 elements, which were then installed within the silicon tubing (inner diameter × length: 4.8 mm × 6.0 cm). A series of blade elements with a 90° crossing angle were arranged in a right‐left twisted manner, with each blade element twisted at an angle of 180°. To perform CDT coagulation under static conditions, the citrated platelet‐poor plasma was recalcified with 6.5 mg mL^−1^ CaCl^2^ and subsequently filled with the helical blade‐mounted tubing. Under flow conditions, 5 mL of platelet‐poor plasma was circulated through the channel containing the blade‐mounted tubing at a flow rate of 20 mL h^−1^ using a peristaltic pump (Ismatec Reglo ICC Independent‐Channel Control Peristaltic Pump, ISMATEC, Wertheim, Germany). Concurrently, 6.5 mg mL^−1^ CaCl^2^ solution was continuously injected at the tubing's inlet, along with a 20 mg mL^−1^ citrate solution. The calcium ion concentration in platelet‐poor plasma before coagulation is maintained at <0.1 mmol L^−1^ using citrate, and it is adjusted to 1.2–1.3 mmol L^−1^ during coagulation by controlled injection of a calcium chloride solution. A thrombus began to form in each element of the helical blade structure as the platelet‐poor plasma flowed and was fully established within an hour, after which the flow was halted.

### Computational Fluid Dynamics Simulation

To predict the trajectory and attachment rate of bacterial cells within the flow channel of the eCDTF, COMSOL Multiphysics software (COMSOL Inc., MA, USA) was utilized. The simulations accounted for half of the flow path's geometric length, as detailed in Movie S1 (Supporting Information). The particle diameter was standardized to 1 µm, consistent with the dimensions of *S. aureus*. These particles were introduced into the flow channel at a velocity of 2.2 mm s^−1^ and were designed to freeze upon adhering to the internal surface, utilizing the Physics of Particle Tracing for Fluid Flow.

### Micro‐CT Scanning

Prior to scanning, the flow path within the eCDTF must be saturated with a contrast solution to ensure the flow path's visibility. Vascupaint (MediLumine, Quebec, Canada) was introduced into the eCDTF at a flow rate of 20 mL h^−1^ until the entire flow path was filled, utilizing a syringe pump (Fusion 200, Chemyx Inc, TX, USA). Once prepared, eCDTF saturated with the contrast solution was imaged using a micro‐CT scanner (SkyScan 1176, Bruker, MA, USA). Subsequently, 3D images were generated with the Micro‐CT Volume Rendering Software (CTVox, Bruker, MA, USA).

### Scanning Electron Microscope (SEM) Imaging

A portion of the CDT was extracted from one section of the helical blades and then cleansed with saline before being fixed in a 2.5% glutaraldehyde solution for an hour. Following triple saline washes, the specimen underwent a graded dehydration process involving successive ethanol baths of increasing concentrations (25%, 50%, 75%, 95%, and 100%). This was followed by a dehydration step using a 1:1 mixture of hexamethyldisilane (HMDS) and ethanol and a final treatment with pure HMDS to thoroughly dry the specimen. Prior to imaging, the specimens were coated with a thin layer of gold‐palladium (20 mA for 60 s) using an ion sputter coater (MC 1000 Ion Sputter Coater, Hitachi High‐Tech, Tokyo, Japan). Imaging was conducted with the Cold Type FE‐SEM (S‐4800, Hitachi High‐Tech, Tokyo, Japan).

### eCDTF Hemoadsorption In Vitro

Prior to operating the extracorporeal filter, bacteria at a concentration of 10^5^ CFU mL^−1^ were introduced into 20 mL of citrate‐anticoagulated human whole blood contained in a 50 mL conical tube. The blood reservoir was subjected to continuous agitation using a thermomixer (Eppendorf Thermomixer C, Eppendorf, Oldenburg, Germany) set to a cycle of 300 rpm for 15 s, followed by a 15‐s pause, all maintained at 4 °C. The bacteria‐inoculated blood was then circulated through the eCDTF at a flow rate of 20 mL h^−1^ using a peristaltic pump over a 3‐h duration (Figure S8, Supporting Information). The flow rate of 20 mL h^−1^ was chosen based on the previously optimized flow velocity (2.2 mm s^−1^), as illustrated in Figure 2H. As a control group, extracorporeal circulation without eCDTF was conducted to validate the bacterial depletion efficiency of eCDTF in whole blood (Blank circulation in Figure 5). During a 3‐h circulation, 100 µL blood samples were retrieved from the channel's outlet every 30 min. Post dilution with 1×PBS (1:10 ratio), the samples were plated on LB agar. The plates were then incubated overnight at 37 °C to cultivate bacterial colonies. The variance in bacterial numbers during the cleansing process was quantified by counting the colonies grown on the LB agar.

To evaluate the efficiency of LPS removal, 200 µg of LPS was added to 20 mL of human whole blood samples. These samples were then processed through the in‐vitro blood‐cleansing system, as detailed above. LPS concentrations were determined using an ELISA kit (LS‐F55757‐1, LSBio, WA, USA).

### Rat Bacteremia Models

All animal procedures received IACUC approval from UNIST (UNISTIACUC‐22‐70). Male Wistar rats, 8 weeks old (Orient Bio Inc, Seongnam, South Korea), were anesthetized by inhalation of 4% isoflurane (Kyongbo Pharm, Seoul, South Korea). Rat jugular vein catheters (SAI‐Infusion, IL, USA) were utilized for catheterization. Throughout the anesthetization, vital parameters such as heart rate, SpO2, rectal temperature, and respiratory rate were diligently monitored using a physiological system (Harvard Apparatus, MA, USA). To establish an MRSA bacteremia model in the rats, 500 µL of 0.9% normal saline (HK inno.N, Seoul, South Korea), which carried 5 × 10^9^ CFU of MRSA, was administered intravenously via the jugular vein catheter.

### In Vivo Extracorporeal Blood‐Cleansing Treatment of Rats

Rat bacteremia models, developed using the previously described protocol^[^ ^47^ ^]^ served to assess the therapeutic efficacy of CDT hemoadsorption in vivo. The inlet of the extracorporeal blood‐cleansing circuit was connected to one of the jugular vein catheters, while the outlet was linked to another catheter. A peristaltic pump circulated the blood at a flow rate of 20 mL h^−1^ for 3 h. To mitigate thrombogenesis during blood cleansing, heparin was continuously introduced into the circuit at a rate of 16 U h^−1^ through a 3‐way valve (Masterflex, IL, USA). Blood samples (30 µL) were drawn every hour to gauge bacterial concentrations. For comprehensive evaluations, blood quantities of 200 µL, 400 µL, 400 µL, and 200 µL were taken immediately before and after the extracorporeal circulation to analyze complete blood count (CBC), cytokine levels, TAT levels, and hemolysis indicators, respectively. Following the initial 3‐h treatment, the rats returned to their cages to recuperate, mitigating excessive anesthesia risks for subsequent treatments. The second 3‐h blood‐cleansing treatment was executed 24 h after the commencement of the initial treatment. Throughout a 7‐day observation period, the survival and health statuses of untreated and eCDTF‐treated rats were systematically monitored every 8 h. After the observation period, rats were humanely euthanized in compliance with IACUC protocols.

Whole blood samples were processed to analyze the counts of RBCs, WBCs, and platelets using a CBC machine (VetScan HM5, ABAXIS, Zoetis, UK). Platelet‐poor plasma samples, extracted from centrifugation at 800 × *g* for 15 min, were utilized for deeper analysis. Cytokine levels were examined via the Bioplex Pro Rat cytokine plex assay (Bio‐Rad, CA, USA),^[^ ^83^ ^]^ while TAT concentrations were determined using an ELISA (Rat TAT complexes ELISA Kit, NOVUS Bio, CO, USA). Hemolysis extents were gauged by absorbance measurements of platelet‐poor plasma at a 540 nm wavelength using a Synergy Neo2 HTS Multi‐Mode Microplate Reader (BioTek, VT, USA).

### Immunohistochemistry

The lungs, spleens, and kidneys were extracted from the euthanized rats. These organs underwent fixation in a 4% paraformaldehyde solution before being embedded in paraffin. Subsequently, these paraffin‐embedded samples were sectioned using a microtome (Leica Microsystems, Wetzlar, Germany). For immunofluorescence staining, the tissue sections were deparaffinized, rehydrated with deionized water, and subjected to antigen retrieval in 10 × 10^−3^ m citrate buffer. Following a blockage with a solution of 2% BSA and 10% goat serum, the tissues were incubated overnight at 4 °C with an anti‐*S. aureus* antibody (Abcam), diluted 1:100 in the blocking solution, targeting MRSA that had infiltrated the organs. The tissue sections were then treated with the corresponding goat anti‐rabbit Alexa Fluor 488 antibody for 2 h. DAPI was employed for nuclear staining. Visualizations were captured using a confocal laser microscope (LSM980, Zeiss, Oberkochen, Germany).

### Statistical Analysis

All data are presented as mean ± S.E.M. Statistical significance between groups was assessed using a two‐tailed Student's t‐test or analysis of variance (ANOVA) followed by Dunnett's post hoc analysis. A *p*‐value of <0.05 was considered statistically significant. All statistical analyses were conducted using GraphPad Prism (Version 8, GraphPad Software Inc., CA, USA) and Origin (OriginLab, MA, USA). Sample sizes (*n*) for each statistical analysis are specified in the corresponding figure legends.

## Conflict of Interest

The authors declare no conflict of interest.

## Author contributions

B.H.J. and J.H.K. designed the study and developed the theoretical model. B.H.J. and S.H.J. fabricated the devices and prepared the blood samples. B.H.J. performed the in vitro experiments. B.H.J. and S.K. prepared the animal models for in vivo experiments. B.H.J. and S.J.P. conducted in vivo experiments. B.H.J. prepared and conducted the high‐speed imaging. B.H.J. and J.H.K. wrote the manuscript. J.H.K. supervised the project and provided critical revisions to the manuscript.

## Supporting information



Supporting Information

Supplemental Movie 1

Supplemental Movie 2

## Data Availability

The data that support the findings of this study are available from the corresponding author upon reasonable request.
