# Supplementary material for: Red Blood Cell‐Induced Bacterial Margination Improves Microbial Hemoadsorption on Engineered Cell‐Depleted Thrombi, Restoring Severe Bacteremia in Rats
Source: Adv Sci (Weinh). 2025 Apr 26;12(29):2417498. doi: 10.1002/advs.202417498 (PMC12362807; doi:10.1002/advs.202417498)
Supplement: Supplementary file 1 — Supporting Information [file ADVS-12-2417498-s003.docx]

**Supporting Information**

**Red Blood Cell-Induced Bacterial Margination Improves Microbial Hemoadsorption on Engineered Cell-Depleted Thrombi, Restoring Severe Bacteremia in Rats**

*Bong Hwan Jang, Su Hyun Jung, Seyong Kwon, Sung Jin Park, and Joo H. Kang**

This file includes

Figure S1 to S8, Table S1, and Note S1.

**
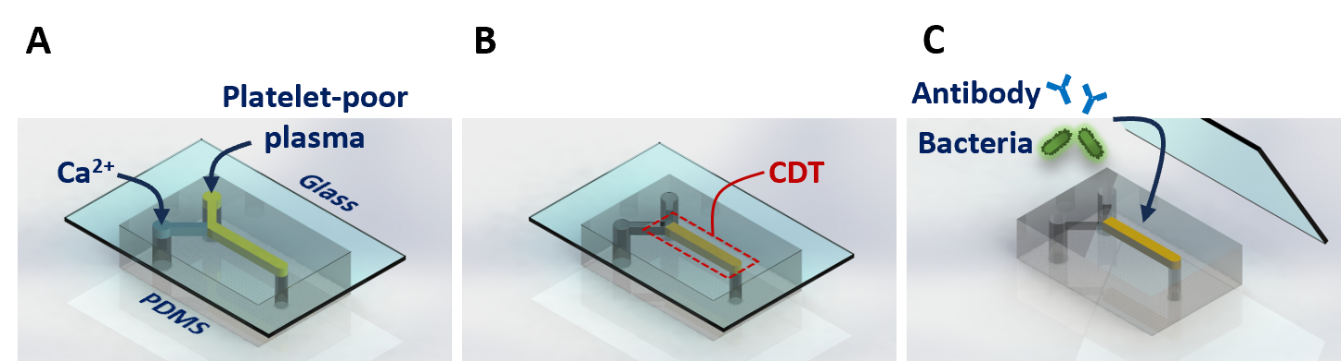
**

**Figure S1. Schematic illustration of cell-depleted thrombus (CDT) fabrication for the microscopic examination of adhesin receptor proteins.** A) Platelet-poor plasma and calcium solution were concurrently introduced into the microfluidic channel. B) The mixture of platelet-poor plasma and ionized calcium underwent continuous coagulation within the channel. C) After removing the glass slide, the CDT was retained within the microfluidic channel. Subsequently, bacteria or antibodies were applied to the CDT surface to facilitate the microscopic observation of plasma proteins functioning as adhesin receptors.


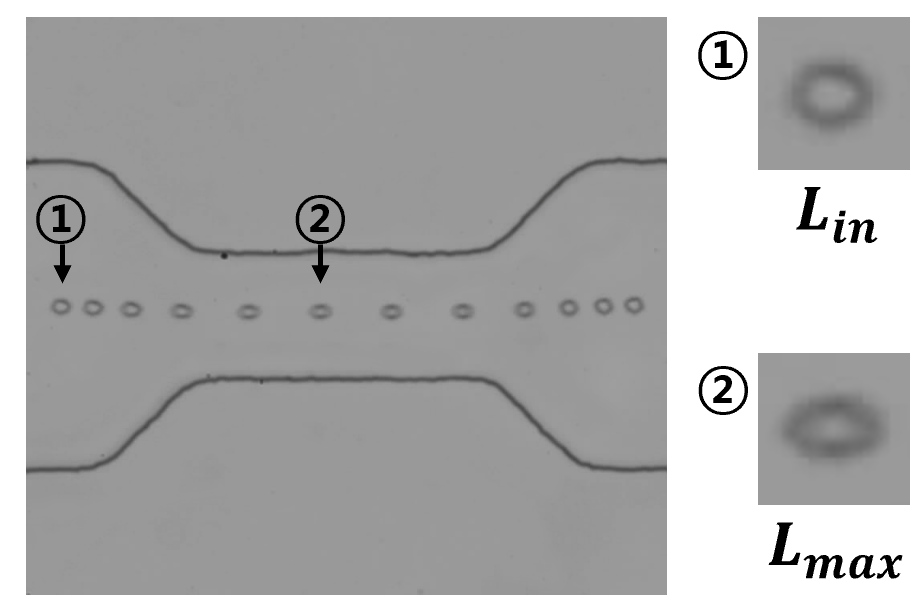


**Figure S2.** **Time-lapse imaging of RBC deformation during flow through a narrow channel.** The deformability of red blood cells (RBCs) was assessed by comparing their maximum elongated length ($L_{max}$) within the narrow channel to their initial length ($L_{in}$).


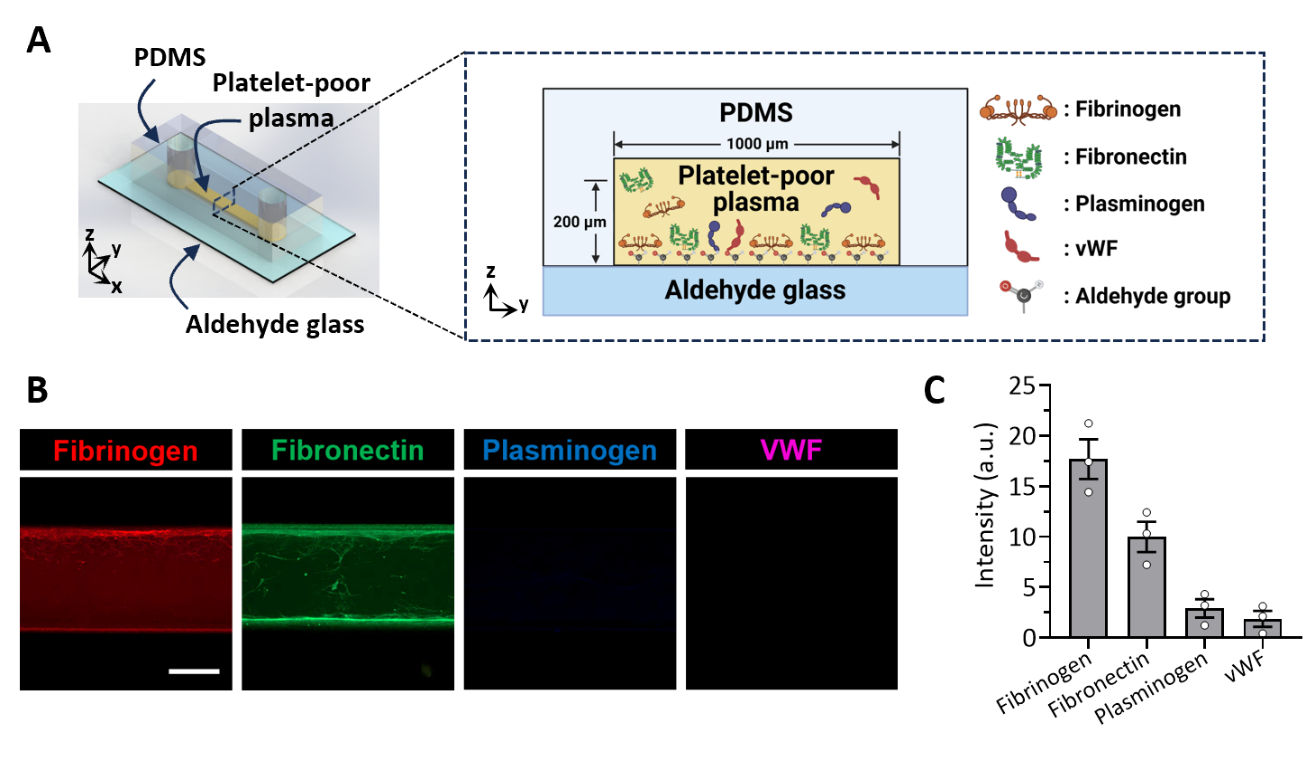


**Figure S3. CDT-coated microfluidic platform for bacterial adhesion assessment.** A) Plasma proteins from platelet-poor plasma were continuously conjugated to the aldehyde groups on the surface of aldehyde-treated glass, forming the CDT-coated surface. B) Immunofluorescence staining of receptor proteins on the CDT-coated surface, highlighting the presence of fibrinogen (red), fibronectin (green), plasminogen (blue), and von Willebrand factor (vWF) (violet). Scale bar: 200 µm. C) The component proportion ratio of the CDT-coated surface closely matches that of the coagulated CDT surface. Values are presented as the mean ± S.E.M. Statistical significance was calculated by a two-tailed Student’s t-test. **p* < 0.05; ***p* < 0.01; ****p* < 0.001; NS, not significant.


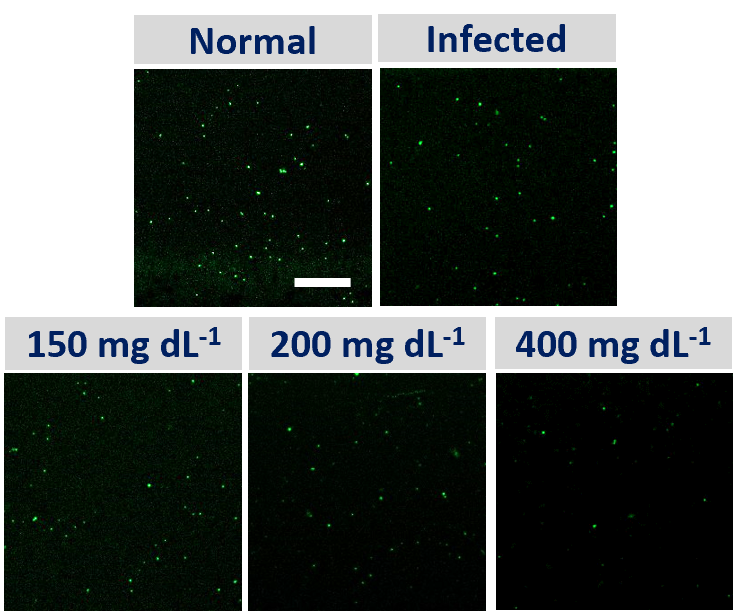


**Figure S4. Evaluation of bacterial adhesion to the CDT-coated surface under different RBC deformability conditions induced by varying glucose concentrations in whole blood.** Hyperglycemic blood was prepared by adding 50-300 mg dL^-1^ glucose to whole blood. Fluorescent images showing the adherence of bacteria (MRSA, green) to the CDT-coated surface after the flow of normal and hyperglycemic blood. Scale bar: 50 µm.


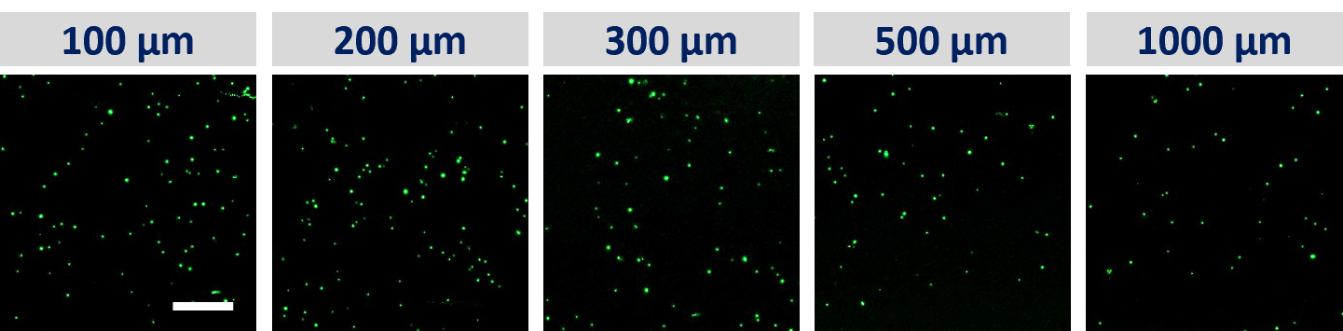


**Figure S5. Evaluation of bacterial adhesion to the CDT-coated surface at different microfluidic channel heights.** Fluorescent images showing bacteria (methicillin-resistant *Staphylococcus aureus* (MRSA), green) adhering to the CDT-coated surface after the flow of bacteremic blood through channels with heights of 100, 200, 300, 500 and 1000 µm. Scale bar: 50 µm.


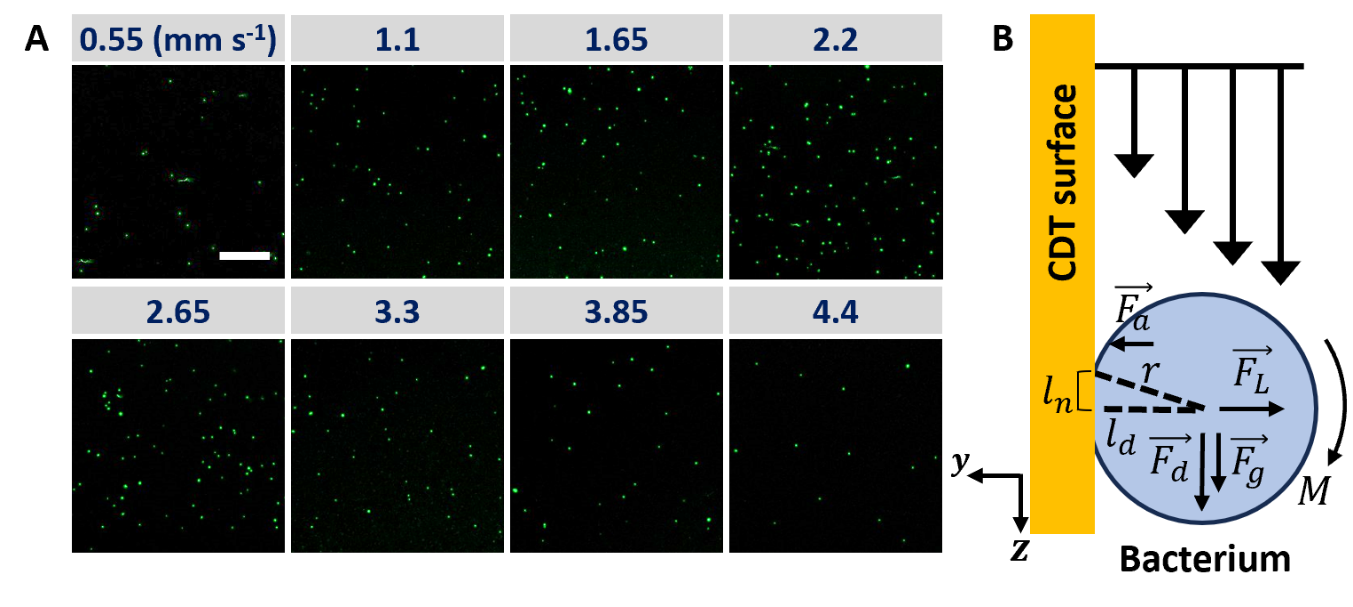


**Figure S6. Evaluation of bacterial adhesion to the CDT-coated surface across different conditions of flow velocity.** A) Fluorescent images displaying bacteria (MRSA, green) adhered to the CDT-coated surface after the flow of bacteremic blood through channels with varied flow velocity (0.55 ~ 4.4 mm s^-1^). Scale bar: 50 µm. B) Torque balance for a spherical bacterium adhered to the CDT surface under shear flow. The four forces acting on the particle are drag ($\vec{F_{d}}$), lift ($\vec{F_{L}}$), adhesion force ($\vec{F_{a}}$), and gravity ($\vec{F_{g}}$). $M$ is the moment exerted by drag. The four forces generate two opposing torques at pivots corresponding to the drag lever arm ($l_{d}$) and normal lever arm ($l_{n}$), respectively.


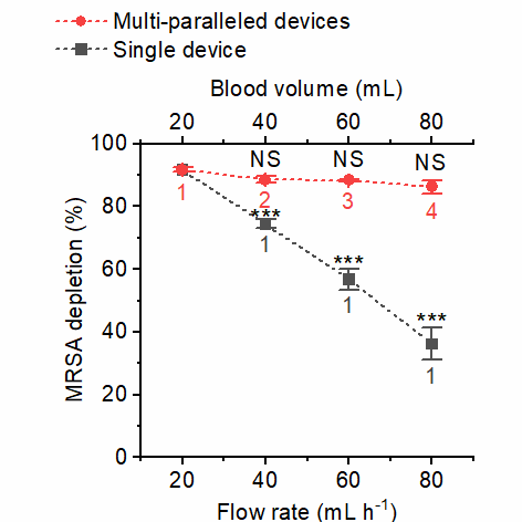


**Figure S7. Comparative evaluation of MRSA depletion rate using multiple parallel eCDTFs compared to a single eCDTF.** The number of multi-paralleled devices increased proportionally to the flow rate and blood volume to sustain bacterial removal efficiency near 90%. Conversely, a single device exhibited a decline in bacterial removal efficiency as the flow rate and blood volume increased. Values are presented as the mean ± S.E.M. Statistical significance was calculated by a two-tailed Student’s t-test. **p* < 0.05; ***p* < 0.01; ****p* < 0.001; NS, not significant.


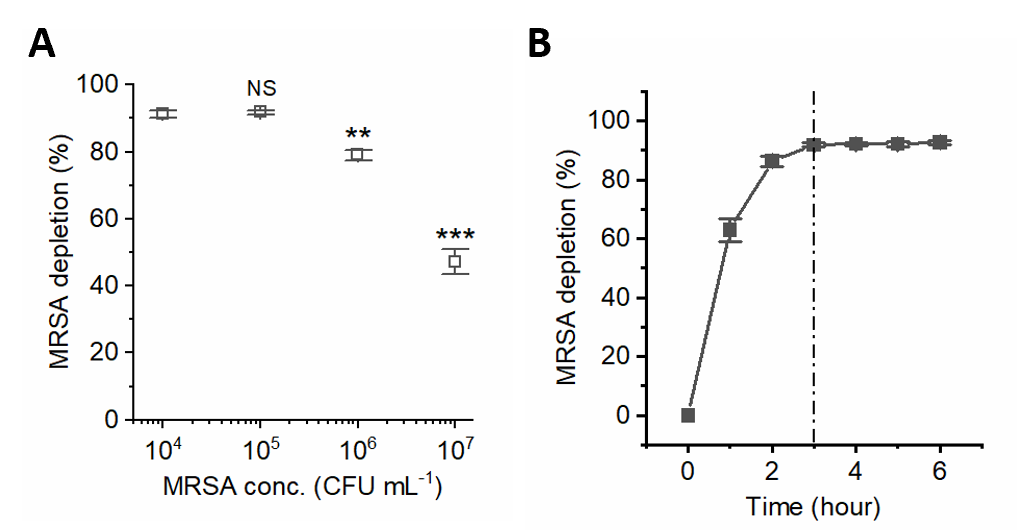


**Figure S8. Assessment of bacterial removal capacity and function maintenance time of eCDTF.** A) A comparative evaluation of the MRSA depletion rate using eCDTF across MRSA concentrations of 10^4^, 10^5^, 10^6^, and 10^7^ CFU mL^-1^. The eCDTF achieved a consistent removal efficiency of approximately 90% at 10^4^ and 10^5^ CFU mL^-1^, while efficiency declined to below 80% at concentrations of 10^6^ and 10^7^ CFU mL^-1^. B) Assessment of the MRSA removal efficiency by eCDTF at hourly intervals over a 6-hour blood-cleansing operation, showing that the removal efficiency approaches the 90% depletion rate threshold after 3 hours of operation. Values are presented as the mean ± S.E.M. Statistical significance was calculated by a two-tailed Student’s t-test. **p* < 0.05; ***p* < 0.01; ****p* < 0.001; NS, not significant.

**Table S1. List of bacterial species in human fecal material inoculated into whole blood and the distribution of the depletion rate for each species.**

| **Depletion rate (%)** | **Bacteria species** |
| --- | --- |
| **100**  **100** | *Bifidobacterium catenulatum* |
|  | *Bifidobacterium pseudocatenulatum* |
|  | *Colidextribacter massiliensis* |
|  | *Clostridium porci* |
|  | *Eubacterium ramulus* |
|  | *Anaerostipes hadrus* |
|  | *Anaerotignum aminivorans* |
|  | *Anaerotignum faecicola* |
|  | *Blautia luti* |
|  | *Blautia wexlerae* |
|  | *Butyribacter intestini* |
|  | *Eisenbergiella massiliensis* |
|  | *Hominisplanchenecus faecis* |
|  | *Lachnoclostridium edouardi* |
|  | *Lachnoclostridium urinimassiliense* |
|  | *Lachnospira pectinoschiza* |
|  | *Lacrimispora amygdalina* |
|  | *Lacrimispora sphenoides* |
|  | *Mediterraneibacter butyricigenes* |
|  | *Mediterraneibacter faecis* |
|  | *[Ruminococcus] lactaris* |
|  | *Roseburia faecis* |
|  | *Roseburia intestinalis* |
|  | *Agathobaculum butyriciproducens* |
|  | *Flavonifractor plautii* |
|  | *Marseillibacter massiliensis* |
|  | *Oscillibacter massiliensis* |
|  | *Pseudoflavonifractor gallinarum* |
|  | *Ructibacterium gallinarum* |
|  | *Vescimonas coprocola* |
|  | *Dialister invisus* |
|  | *Bacteroides eggerthii* |
|  | *Bacteroides thetaiotaomicron* |
|  | *Alistipes shahii* |
|  | *Parabacteroides distasonis* |
|  | *Paracoccus aeridis* |
|  | *Sutterella megalosphaeroides* |
|  | *Shigella flexneri* |
| **90 ~ 99.99** | *Bacteroides uniformis* |
|  | *Faecalibacterium prausnitzii* |
|  | *Roseburia inulinivorans* |
|  | *Waltera intestinalis* |
|  | *Phocaeicola vulgatus* |
|  | *Phocaeicola coprocola* |
|  | *Bacteroides stercoris* |
|  | *Lachnospira eligens* |
|  | *Agathobacter rectalis* |
|  | *Coprococcus comes* |
|  | *Paraprevotella clara* |
|  | *Leyella stercorea* |
|  | *Bacteroides ovatus* |
|  | *Phocaeicola plebeius* |
|  | *Parabacteroides merdae* |
|  | *Gemmiger formicilis* |
|  | *Megamonas rupellensis* |
|  | *Blautia obeum* |
|  | *Bacteroides xylanisolvens* |
|  | *Faecalibacterium longum* |
|  | *Alistipes putredinis* |
|  | *Phocaeicola coprophilus* |
|  | *Lactobacillus rogosae* |
| **60 ~ 89.99** | *Bacteroides humanifaecis* |
|  | *Lacrimispora indolis* |
|  | *Bilophila wadsworthia* |
|  | *Faecalibacterium duncaniae* |
|  | *Blautia phocaeensis* |
|  | *Faecalibacterium hattorii* |
|  | *Konateibacter massiliensis* |
|  | *Faecalibacillus faecis* |
|  | *Gluceribacter canis* |
|  | *Fusicatenibacter saccharivorans* |
|  | *Enterocloster bolteae* |
|  | *Faecalibacillus intestinalis* |
|  | *Corynebacterium dentalis* |
|  | *Dorea phocaeensis* |
|  | *Eubacterium ventriosum* |

**Note S1. Flow-driven detachment of bacterial cells from the cell-depleted thrombus surface**

**1. Analyzing forces acting on a bacterium adhered to the cell-depleted thrombus surface**

We investigated the adhesion dynamics of bacteria on the surface of the cell-depleted thrombi (CDT) in response to changes in flow velocity. With a vertical approach to bacteria delivery onto this surface, one would anticipate increased bacterial adhesion as the flow velocity of infected blood rises. Intriguingly, our data indicated that bacterial adhesion starts to wane once the flow surpasses 2.2 mm s^-1^. To understand this observation, we calculated the forces acting on the adhered bacterial cell. Four primary forces influence a bacterium adhered to a CDT surface: hydrodynamic forces, specifically drag ($\vec{F_{d}}$) and lift ($\vec{F_{L}}$), the adhesive force ($\vec{F_{a}}$), and gravitational force ($\vec{F_{g}}$) (Figure S6B).^[1,2]^ In conditions where infected blood flows through the channel, the flow direction is parallel to the gravitational force. This setup is analogous to observations made during extracorporeal CDT hemoadsorption. The bacterium, identified as *Staphylococcus aureus (S. aureus)*, was represented as a sphere with a diameter of 1 µm.

***1.1. Gravitational force***

The gravitational force acting on a bacterium can be defined by:

$\vec{F_{g}}=\frac{4}{3}\pi r^{3}g\left( \rho_{b}-\rho\right)\hat{z}$ (1)

where $r$ is the radius of a bacterium, $g$ is the acceleration of gravity, $\rho_{b}$ is the density of a bacterium, and $\rho$ is the density of the platelet-poor plasma.

***1.2. Hydrodynamic forces (drag and lift)***

Hydrodynamic forces act to detach the particles from the CDT surface. The drag and lift forces for a sphere are given as:

$\vec{F_{d}}=10.2054\pi\mu r^{2}\gamma\hat{z}$ (2)

$\vec{F_{L}}=-9.22r^{4}\gamma^{2}\rho\hat{y}$ (3)

where $\mu$ is the dynamic viscosity of platelet-poor plasma and $\gamma$ is the shear rate.

***1.3. Adhesion force***

Surface adhesin proteins on bacteria, including ClfA and FnBPB, facilitate bacterial adherence to biomaterials coated with plasma proteins, thereby enabling bacterial colonization and biofilm formation. Prior research employing atomic force microscopy (AFM) has quantified the bond strength between these adhesins and their receptors. When subjected to minimal tensile forces, bond strengths approximately range from 50 to 150 pN.^[3,4]^ We utilized this range of adhesion force to predict conditions required for the detachment of a bacterium.

**2. Flow-driven bacterial detachment conditions**

We studied the conditions under which a spherical bacterium detaches from a CDT surface. The factors influencing the bacterial detachment are a balance of drag ($F_{d}$), lift ($F_{L}$), gravitational ($F_{g}$), and adhesion ($F_{a}$) forces acting upon the bacterium. At the point of contact between the bacterium and the substrate, the normal forces can induce a minor deformation, resulting in the normal lever arm ($l_{n}$) (Figure S6B). By applying the equilibrium of forces and torques (EFT) principle, there are three potential detachment mechanisms for the bacterium: lifting, sliding, and rolling, as delineated in expressions (1), (2), and (3), respectively. A disruption in any one of these equilibria implies imminent detachment.

(Lifting) $F_{L}> F_{a}$ (4)

(Sliding) $F_{d} + F_{g} > \mu_{s}(F_{a}- F_{L})$ (5)

(Rolling) $M + F_{L}l_{n} > F_{a}l_{n}$ (6)

***2.1. Lifting***

For flow velocities ranging from 0.55 ~ 4.40 mm s^-1^ in a channel with a height of 200 µm, the lift force $F_{L}$ is computed to be between 1.00 and 8.02 × 10^-26^ N. Given that $F_{L}$ is considerably smaller than the adhesion force $F_{a}$ (i.e., $F_{L}\ll F_{a})$, the possibility of detachment via lifting can be dismissed.

***2.2. Sliding***

At flow velocities between 0.55 and 4.40 mm s^-1^, the calculated drag force $F_{D}$ is in the range of 2.20 to 17.62 × 10^-13^ N. For a bacterium with a diameter of 1 µm, the gravitational force is determined to be 3.85 × 10^-16^ N. In earlier research, the frictional coefficient for clots with 0% RBC was found to be approximately 0.5.^[5]^ Considering that the combined term $F_{d} + F_{g}$ is less than $\mu_{s}(F_{a}- F_{L})$, the potential for detachment through sliding is unlikely.

***2.3. Rolling***

In evaluating the terms in equation (6), $F_{L}l_{n}$ can be neglected since $F_{L}$ is significantly smaller than the adhesion force $F_{a}$ (i.e., $F_{L}\ll F_{a})$. $M$ denotes the moment produced by the drag, transmitted through the drag lever arm $l_{d}$. For a sphere, the moment $M$ is defined by:

$M= 1.37F_{d}l_{d} (l_{d}= \sqrt{r^{2}-{l_{n}}^{2}})$ (7)

Using the Hertz contact theory, the normal lever arm $l_{n}$ can be derived, which aids in determining the contact deformation area’s radius. With an effective Young’s modulus ($E_{Y}$) for a sphere, $l_{n}$ is given as:

$l_{n}= \sqrt[3]{\frac{3\left( F_{d}+F_{g}-F_{L} \right)r}{4E_{Y}}}$ (8)

The moment attributed to adhesion $F_{a}l_{n}$ falls within the range of 10.92 and 39.04 × 10^-19^ $kg\cdot m^{2}/s^{2}$. At an optimized linear flow velocity of 2.2 mm s^-1^ as depicted in Figure 2H, the drag moment $M$ approximates 6.03 × 10^-19^ $kg\cdot m^{2}/s^{2}$, positioning it below the minimum of adhesion moment range. Beyond this velocity, the drag moment $M$ begins to surpass the minimum moment of adhesion $F_{a}l_{n}$, meeting the criteria for rolling detachment. Based on this theoretical analysis, we postulate that the observed decrease in bacterial attachment at flow velocities exceeding 2.2 mm s^-1^, as shown in Figure 2H, can be attributed to rolling detachment driven by the increased drag force.

**3. References**

[1] M. A. Hubbe, *Colloids and Surfaces* **1984**, *12*, 151.

[2] H. Z. Ting, P. Bedrikovetsky, Z. F. Tian, T. Carageorgos, *Chem Eng Sci* **2021**, *241,* 116658.

[3] T. M. da Costa, A. Viljoen, A. M. Towell, Y. F. Dufrêne, J. A. Geoghegan, *Nat Commun* **2022**, *13*, 2517.

[4] P. Herman-Bausier, C. Labate, A. M. Towell, S. Derclaye, J. A. Geoghegan, Y. F. Dufrêne, *Proc Natl Acad Sci U S A* **2018**, *115*, 5564.

[5] G. M. Gunning, K. McArdle, M. Mirza, S. Duffy, M. Gilvarry, P. A. Brouwer, *J Neurointerv Surg* **2018**, *10,* 34.

**Description of Additional Supporting Information**

**File Name: Movie S1**

**Description:** Particle tracing simulations conducted using COMSOL Multiphysics® software, showcasing particles freezing upon contact with the inner surfaces of a channel without helical blades.

**File Name: Movie S2**

**Description:** Particle tracing simulations conducted using COMSOL Multiphysics® software, showcasing particles freezing upon contact with the inner surfaces of a channel with helical blades.
